# QKI degradation in macrophage by RNF6 protects mice from MRSA infection via enhancing PI3K p110β dependent autophagy

**DOI:** 10.1186/s13578-022-00865-9

**Published:** 2022-09-10

**Authors:** Dongsheng Zhai, Wenwen Wang, Zichen Ye, Ke Xue, Guo Chen, Sijun Hu, Zhao Yan, Yanhai Guo, Fang Wang, Xubo Li, An Xiang, Xia Li, Zifan Lu, Li Wang

**Affiliations:** 1 Department of Pharmacology, School of Pharmacy, Fourth Military Medical University, Xi’an, Shaanxi China; 2 State Key Laboratory of Cancer Biology, Department of Biopharmaceutics, Fourth Military Medical University, Xi’an, Shaanxi China; 3 Air Force Health Service Training Base of PLA, Fourth Military Medical University, Xi’an, Shaanxi China; 4 Department of Dermatology, Xijing Hospital, Fourth Military Medical University, Xi’an, Shaanxi China; 5 State Key Laboratory of Cancer Biology, Department of Pharmacogenomics, School of Pharmacy, Fourth Military Medical University, Xi’an, Shaanxi China; 6 State Key Laboratory of Cancer Biology and National Clinical Research Center for Digestive Diseases, Xijing Hospital of Digestive Diseases, Fourth Military Medical University, Xi’an, Shaanxi China; 7 Department of Microbiology, Fourth Military Medical University, Xi’an, Shaanxi China; 8 State Key Laboratory of Cancer Biology, Department of Biochemistry and Molecular Biology, the Fourth Military Medical University, Xian, 710032 Shaanxi China

**Keywords:** Quaking, Macrophage, MRSA, Sepsis, Autophagy, PI3K-p110β, P body

## Abstract

**Background:**

Sepsis is a fatal condition commonly caused by Methicillin-resistant *Staphylococcus aureus* (MRSA) with a high death rate. Macrophages can protect the host from various microbial pathogens by recognizing and eliminating them. Earlier we found that Quaking (QKI), an RNA binding protein (RBP), was involved in differentiation and polarization of macrophages. However, the role of QKI in sepsis caused by pathogenic microbes, specifically MRSA, is unclear. This study aimed to investigate the role of QKI in regulation of host–pathogen interaction in MRSA-induced sepsis and explored the underlying mechanisms.

**Methods:**

Transmission electron microscope and immunofluorescence were used to observe the autophagy level in macrophages. Real-time PCR and western blot were used to analyzed the expression of mRNA and protein respectively. The potential protein interaction was analyzed by iTRAQ mass spectrometry and Immunoprecipitation. RNA fluorescence in situ hybridization, dual-luciferase reporter assay and RNA immunoprecipitation were used to explore the mechanism of QKI regulating mRNA of PI3K-p110β.

**Results:**

The mRNA level of QKI was aberrantly decreased in monocytes and PBMCs of septic patients with the increasing level of plasma procalcitonin (PCT). Then the mice with myeloid specific knockout of QKI was challenged with MRSA or Cecal Ligation and Puncture (CLP). Mice in these two models displayed higher survival rates and lower bacterial loads. Mechanistically, QKI deletion promoted phagocytosis and autophagic degradation of MRSA via activating p110β (a member of Class IA phosphoinositide 3-kinases) mediated autophagic response. QKI expression in macrophages led to the sequestration of p110β in mRNA processing (P) bodies and translational repression. Upon infection, the direct interaction of RNF6, a RING-type E3 ligase, mediated QKI ubiquitination degradation and facilitated PI3K-p110β related autophagic removal of pathogen. The administration of nanoparticles with QKI specific siRNA significantly protected mice from MRSA infection.

**Conclusions:**

This study disclosed the novel function of QKI in the P body mRNA regulation during infection. QKI degradation in macrophage by RNF6 protects mice from MRSA infection via enhancing PI3K-p110β dependent autophagy. It suggested that QKI may serve as a potential theranostic marker in MRSA-induced sepsis.

**Graphical Abstract:**

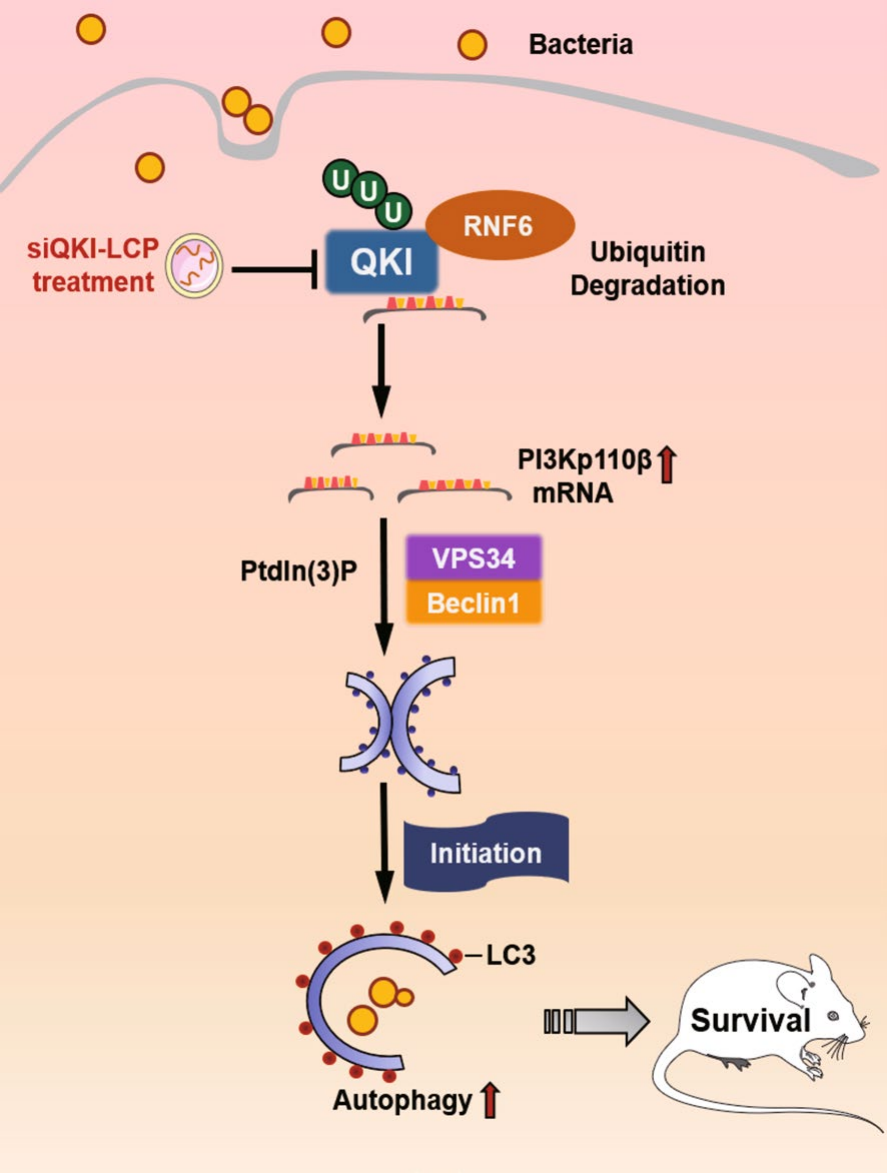

**Supplementary Information:**

The online version contains supplementary material available at 10.1186/s13578-022-00865-9.

## Background

Sepsis presents as an overwhelming immune response towards invasions of pathogenic microbes in host. It manifests as one of the leading causes of mortality in intensive care units (ICUs) [1]. *Staphylococcus aureus*, especially methicillin-resistant *Staphylococcus aureus* (MRSA) has emerged as the main cause of sepsis with the highest fatality rate. In China, annually, 30% of the *S. aureus* infections were due to MRSA. MRSA accounted for 47% of the deaths caused by multi-drug resistant (MDR) bacteria. Therefore, further investigations are required to manage MRSA-induced sepsis [2–4].

Macrophage is a crucial component of innate immune system. It protects the host against various microbial pathogens by recognizing and eliminating them [5]. Accumulating evidences showed that autophagy in macrophage facilitates removals of intracellular pathogens in a process known as xenophagy [6–11]. It employs core autophagy machinery for the initiation and formation of autophagosomes [11]. The members of Class IA phosphoinositide 3-kinases (PI3Ks), PI3Kα and PI3Kδ, activate the PI3K-mTOR pathway and inhibit starvation-induced autophagy [12]. Under the condition of growth factor limitation, PI3Kβ dissociates from growth factor receptor complexes, and increases its interaction with the small GTPase Rab5. The p110β-Rab5 association maintains Rab5 in its GTP-bound state and enhances the Rab5-Vps34 interaction that promotes autophagy [13]. It’s interesting to know which pathway is associated with the xenophagy induction.

P bodies (PBs) are the non-membranous cytosolic organelles involved in RNA metabolisms. Recent innovative isolation methods facilitated the characterizations of its protein and RNA contents [14, 15]. More evidences suggested that PBs were not primarily involved in RNA decay but rather in the coordinated storage of mRNAs encoding regulatory functions. The presence of PBs is important for cell adaption to various environments. There are abundant protein components in the PBs and are broadly categorized into the followings, decapping activators (EDC3, EDC4, PAT1B, LSM1-7), de-adenylation factors (CCR4, PAN3), miRNA-mediated silencing factors (Ago1-4, GW182), nonsense-mediated decay factors (UPF1, SMG5, SMG7), translational repression regulators (DDX6, eIF4E-binding protein 4E-T) and RNA-binding proteins (IGFBP, huR). PB enriched mRNAs are usually encoding regulatory proteins, while PB excluded mRNAs encoding the house-keeping proteins. Therefore, a large variety of cellular functions maybe driven by such dynamic regulations [15].

Quaking (QKI), an RNA binding protein (RBP), which belongs to the signal transduction and activation of RNA (STAR) family, modulated mRNA expressions at the post-transcriptional levels, such as mRNA splicing, mRNA exporting and translational repression [17, 18]. The presence of a KH-family homology domain confers QKI with the capacity to bind target mRNAs specifically to the QKI Response Element (QRE) sequence (NACUAAY N1-20 UAAY) [17]. C/EBPα-activated QKI-5 negatively regulated macrophage differentiation by suppressing CSF1R expression in macrophage progenitor cells [19]. In atherosclerotic lesions, QKI regulated the conversion of monocytes into macrophage cells or foam cells by altering multiple signaling pathways. Large-scale analyses of RNA-sequencing data disclosed that QKI defect may affect multiple target mRNA expressions in the positive or negative ways. However functional related molecular mechanisms remained to be validated. In the previous study, we have shown that QKI modulated macrophage polarization states in LPS-induced endotoxic shock [20]. However, the role of QKI in sepsis caused by pathogenic microbes, specifically MRSA, is worthy to know.

Until now, the in vivo function for QKI in the modulation of macrophage autophagy, especially in host defense against pathogen invasion, has not been elucidated yet. In the present study, we provide evidence that RNF6-mediated QKI degradation protects mice from MRSA infection via enhancing PI3K-p110β dependent autophagy. Our data demonstrated for the first time that QKI could serve as a novel prognostic marker and therapeutic target in sepsis.

## Results

### Myeloid QKI deficiency is protective against MRSA infection

**Fig. 1:**
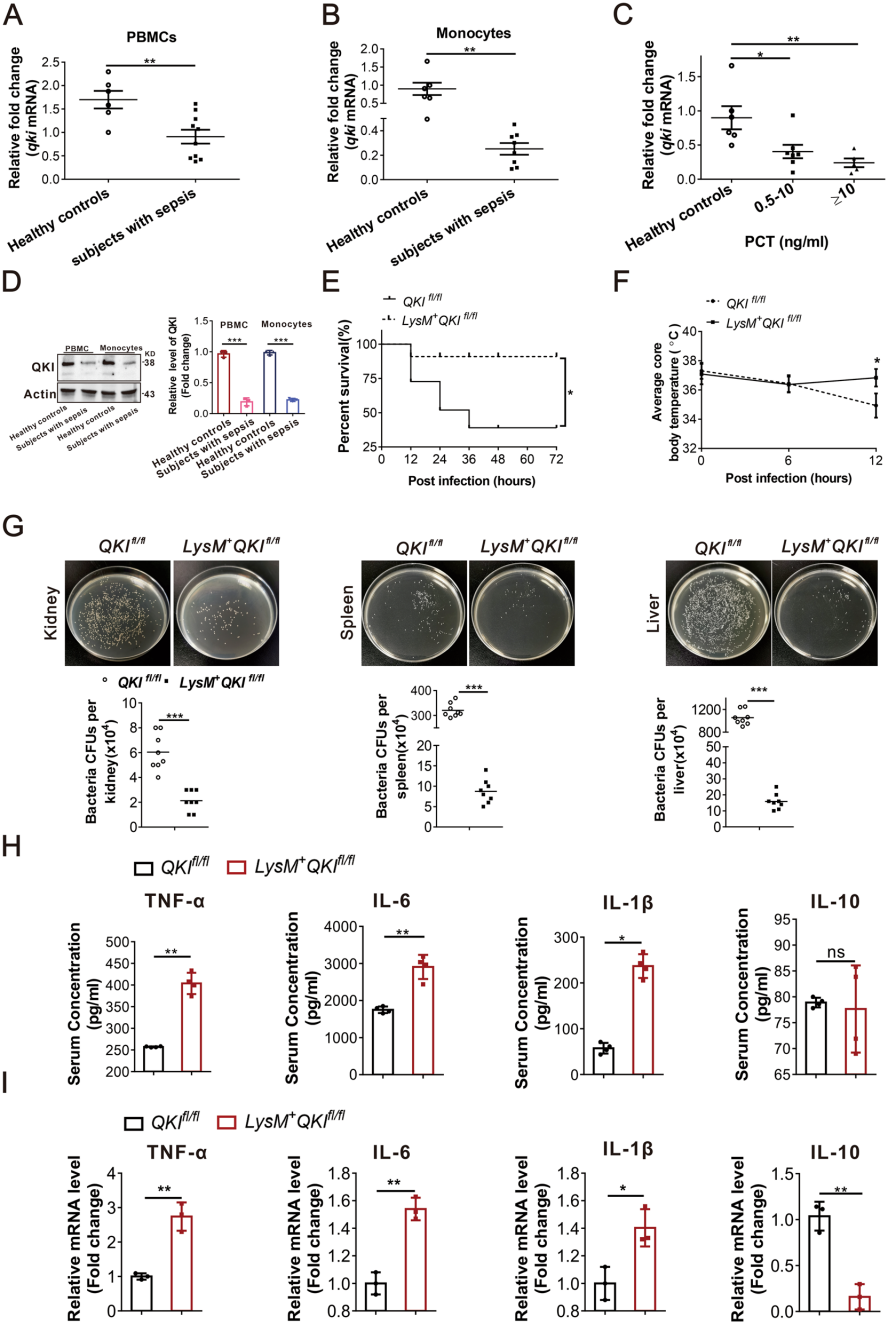
Myeloid QKI deficiency is protective against MRSA infection in vivo. **A** Real-time PCR analysis of relative mRNA expression of QKI in PBMC of the healthy controls and in sepsis patients. **B** Real-time PCR analysis of relative mRNA expression of QKI in monocytes of healthy controls and in sepsis patients. (n = 6 controls and n = 10 sepsis patients). **C** Correlation between the PCT and mRNA expression of QKI in healthy controls and in sepsis patients. **D** Western blot analyzed the expression of QKI in PBMC of the healthy controls and in sepsis patients. (n = 6 controls and n = 6 sepsis patients). One representative immunoblot is shown. Graphs are representative quantification of the band intensity for immunoblots from three independent experiments (right). **E** Mice were injected intraperitoneally with live *S. aureus* (1 × 10^9^ CFU), and the survival rate of mice infected with MRSA within 3 days. **F** The body temperature of mice within 12 h was measured by rectal thermometer after MRSA infection **G** Organs of mice after infection with MRSA 24 h were collected and homogenized. Enumeration of forming bacteria units (c.f.u) from various organs (kidney, spleen, liver) was analyzed. **H** Cytokines (TNF-α, IL-6, IL-1β and IL-10) level in blood were analyzed by ELISA method after infection in 24 h. **I** Real-time PCR analysis of relative mRNA expression of IL-6, IL-10, IL-1β and TNF-α in macrophages (F4/80^+^CD11b^+^) purified by flow cytometry from peritoneal cells after infected with MRSA 24 h. All the bars represented the mean of measurements from three independent experiments, and the error bars indicated ± SD, student’s t test. (**A**–**C**) Healthy controls, n = 6; subjects with sepsis, n = 10. **D**, are representative of three experiments. (**E**–**F**) are from one experiment (n = 12 mice/group). **E**, *p < 0.05 Log-rank (Mantel-Cox) test; **F**, * p < 0.05, (student’s t test). Symbols represent data points for each animal. (**F**) are from one experiment (n = 8 mice/group). (**G**–**H**) are from one experiment (n = 4 mice/group). Symbols represent data points for each animal. *p < 0.05, **p < 0.01. ***p < 0.001 (student’s t test)

We collected blood samples from patients with sepsis meeting SIRS criteria and the healthy controls to study whether QKI was involved in sepsis [22]. Real-time PCR results showed that QKI mRNA expression in peripheral blood mononuclear cells (PBMCs) or monocytes of septic patients was significantly lower than controls (Fig. 1A and B, Additional file 3: Table S1). Furthermore, QKI mRNA levels in peripheral blood monocytes from sepsis patients were decreased with the increasing level of serum procalcitonin (an indicator of the severity of the infection, Fig. 1C). In addition, QKI protein expression was detected. In accord with the mRNA results, the level of QKI protein was decreased in both PBMC and monocytes in sepsis patient (Fig. 1D). These data implicated that the QKI expression was aberrantly reduced in monocytes and PBMCs of septic patients’ blood.

Encouraged by the results from septic patients, we further explored the role of QKI in monocytes/macrophages during sepsis progression using myeloid-specific QKI knockout mice (LysM^+^QKI^fl/fl^). LysM^+^QKI^fl/fl^ mice were created by intercrossing QKI^fl/fl^ mouse with the LysMCre mouse (have a Cre recombinase controlled by a LysM promoter, in which the conditional QKI allele is excised in monocytes, macrophages, and CD11b+ dendritic cells), as our previous reports [19, 23]. Furthermore, survival analysis was performed to investigate the role of QKI in *S. aureus* and CLP (cecal ligation puncture) induced septic shock. LysM^+^QKI^fl/fl^ mice showed a significantly higher survival rate than the control mice (Fig. 1E and Additional file 1: Figure S1A). Also, LysM^+^QKI^fl/fl^ mice were protected from *S. aureus* and CLP induced hypothermia (Fig. 1F and Additional file 1: Figure S1B). Additionally, these mice showed significantly lower bacterial counts in different organs, such as kidney, spleen, and liver (Fig. 1G and Additional file 1: Figure S1C–D). The reduced bacterial count was accompanied by elevated levels of pro-inflammatory cytokines in serum, such as TNF-α, IL-6, and IL-1β in LysM^+^QKI^fl/fl^ mice (Fig. 1H and Additional file 1: Figure S1E). In contrast, levels of anti-inflammatory cytokine IL-10 were identical in both LysM^+^QKI^fl/fl^ mice and control group in MRSA infection model (Fig. 1H). The similar results were attained in peritoneal macrophages (CD11b^+^F4/80^+^) cells. Elevated pro-inflammatory cytokines (TNF-α, IL-6, and IL-1β) but decreased level of anti-inflammatory cytokine IL-10 was detected in LysM^+^QKI^fl/fl^ mice (Fig. 1I).

### QKI deficiency improved the bactericidal activity of macrophages via enhanced autophagy

**Fig. 2:**
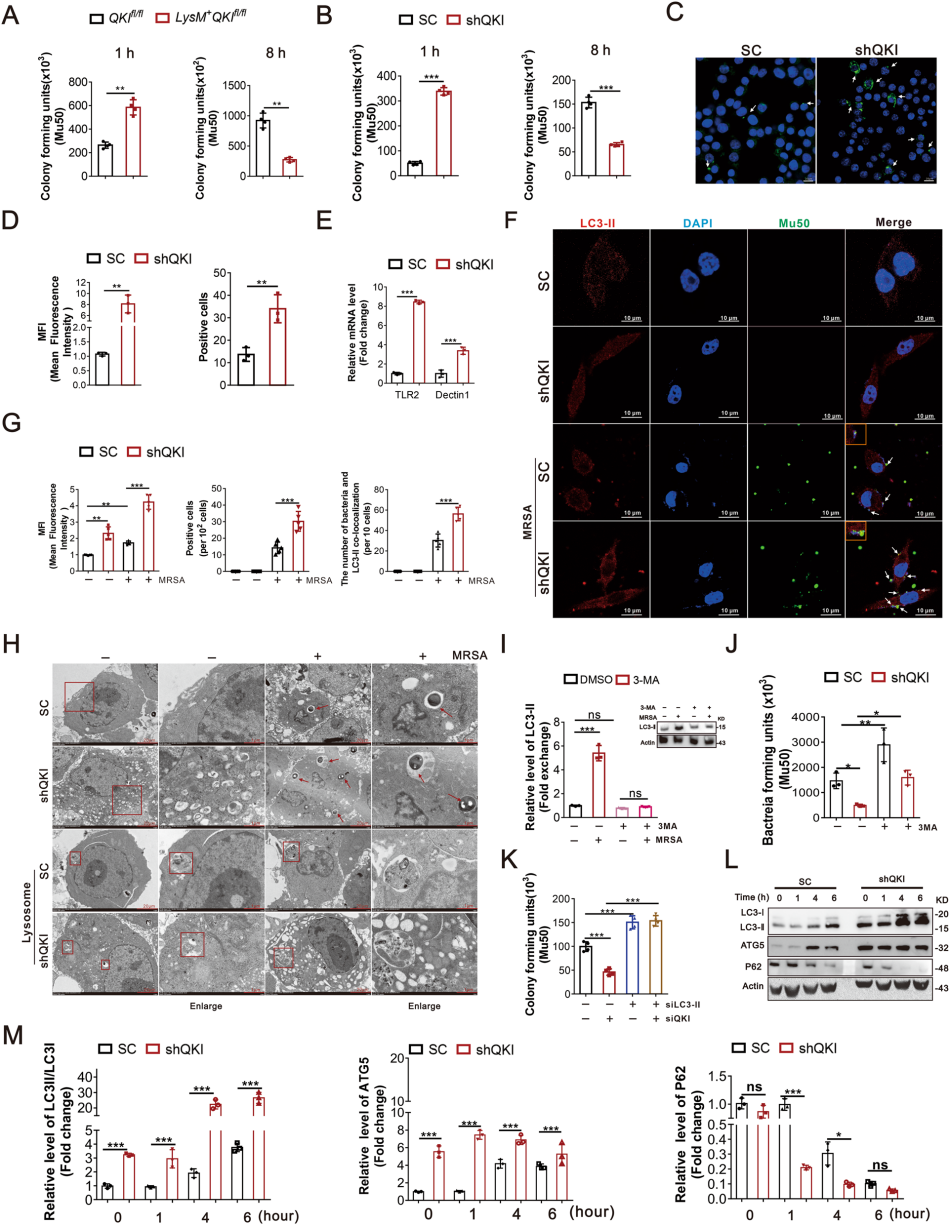
QKI deficiency improved the phagocytic and bactericidal activity of macrophages in vitro. **A** After peritoneal cells were harvested from QKI^fl/fl^ and LysM + QKI^fl/fl^ mice, F4/80^+^CD11b^+^ macrophages were isolated by FACS and incubated with MRSA. At 1 h or 8 h post-incubation, the number of bacteria units forming was counted respectively. Enumeration of forming bacteria units (c.f.u) were shown in the left (1 h) and right (8 h). **B** RAW264.7 cells transfection with shRNA specific for QKI and scrambled shRNA control were incubated with MRSA. The number of bacteria units forming in RAW264.7 cells were calculated and shown after indicated time. **C** Phagocytosis ability was analyzed by phagocytose opsonized particles in RAW264.7 cells after incubated with Latex beads-rabbits IgG-FITC complex. Representative graph of positive area was showed. **D** Graphs showed the quantification of mean fluorescence intensity (MFI) and number of FITC staining positive cells in three randomly fields. **E** Real-time PCR analysis of relative mRNA expression of the receptors level (TLR2 and Dectin-1) in QKI silenced (shQKI) and scramble (SC) RAW264.7 cells after incubating with MRSA. **F** Confocal microscopic imaging of co-localization of FITC-conA stained Mu50 and LC3-II in QKI silenced (ShQKI) and scramble (SC) RAW264.7 cells after MRSA infection. FITC-conA stained Mu50: green, immuno-stained for LC3-II: red, DAPI: blue. (Scale Bar: 10 μm). **G** The quantification of mean fluorescence intensity (MFI) of LC3-II immunostaining was showed (left). The number of FITC staining positive cells in five randomly fields (middle). The number of bacteria and LC3-II co-localization in 10 cells. Five fields were randomly selected to analyzed. **H** The formation of autophagosomes in QKI silenced (ShQKI) and scramble (SC) RAW264.7 cells were observed under transmission electron microscopy. Bacteria within autophagosomes were indicated with thick arrow (Scale Bar: 2 μm). The lysosomes were observed in SC and ShQKI cells, which showed in square box. **I** Western blots were performed to determine the effects of autophagy inhibitor 3-MA with or without MRSA infection. One representative immunoblot is shown (on the left) and the graph (on the right) presents quantification of the band intensity for immunoblots from three independent experiments. The results were shown as expression of LC3-II. **J** “Phagocytosis assay” was used to determine the effect of 3-MA to phagocytosis ability of QKI silenced (ShQKI) and scramble (SC) RAW264.7 cells after treated with MRSA and 3-MA simultaneously. The number of bacteria units forming were calculated after 8 h. Enumeration of forming bacteria units (c.f.u) were shown. **K** LC3-II was knockdown by siRNA, follwed by “Phagocytosis assay” determining the effect of LC3 to phagocytosis ability of QKI silenced (ShQKI) and scramble (SC) RAW264.7 cells. **L** Immunoblot analysis of protein expression of LC3, ATG5 and P62, in scramble and shQKI RAW264.7 cells, with actin as an internal control. One representative immunoblot is shown. **M** Graphs are representative quantification of the band intensity for immunoblots for figure **L**, which was from three independent experiments. All the bars represented the mean of measurements from three independent experiments, and the error bars indicated ± SD. (**A**, **B**, **J** and **L**) are representative of one experiment (**A** and **B**, n = 4 wells/group; J and L, n = 3 wells/group). (**D**) n = 3 fields/group. (**E**) are representative of one experiment (n = 3 wells/group). (**D**) n = 5 fields/group. (b and **L**) are representative of three experiments. *p < 0.05, **p < 0.01. ***p < 0.001, not significant (ns) (student’s t test)

The decreased bacterial burden in myeloid-specific QKI knockout mice suggested enhanced phagocytic and bactericidal activities of macrophages. Peritoneal derived macrophages (CD11b^+^F4/80^+^) were isolated from QKI^fl/fl^ and LysM^+^QKI^fl/fl^ mice and incubated with bacteria. The intracellular bacteria number was counted at 1 h and 8 h post incubation to evaluate the phagocytic and bactericidal ability of macrophages, respectively. The bacterial load was higher at 1 h and lower at 8 h in LysM^+^QKI^fl/fl^ macrophages compared with control indicating that QKI deletion led to enhanced phagocytic and bactericidal activities in macrophages (Fig. 2A and Additional file 2: Figure S2A). Similar findings were observed in RAW264.7 cells transfected with QKI specific siRNA (shQKI) (Fig. 2B and Additional file 2: Figure S2B). Phagocytic activity of macrophages was visualized using the latex beads coated with rabbit IgG-FITC complex, which highlighted the intracellular phagosome. QKI deficient macrophages displayed an enhanced intensity of fluorescent signals (Fig. 2C and D), suggesting enhanced phagocytic activity.

Gram-positive bacteria display multiple phagocytosis-associated ligands. As depicted in Fig. 2E, QKI ablation resulted in increased expression levels of TLR2 and dectin-1 mRNA. FCM analysis showed an elevated level of reactive oxygen species in the QKI deficient macrophages (Additional file 2: Figure S2C). Meanwhile, QKI deficient RAW 264.7 cells showed increased secretion of pro-inflammatory cytokines, such as TNF-α, IL-6, IL-1β, and decreased secretion of IL-10, post-infection (Additional file 2: Figure S2D). These data confirmed that QKI ablation in macrophages facilitated pathogen phagocytosis and removals.

Autophagy is a crucial cellular process of host’s immune defense. It plays a crucial role in antimicrobial response against infectious microbes. Confocal microscopy results (shown in Fig. 2F) displayed that there was co-localization of the red fluorescence (LC3-daylight 594) and green fluorescence (Mu50-FITC) in autophagic vacuoles of RAW264.7 cells. Moreover, the level of autophagy was increased after infection, and there was more co-localization of bacteria and LC3-II immunostaining, indicating the autophagy involved in anti-bacteria process, which was enhanced after QKI deletion (Fig. 2F and G). In addition, as scanning electron microscopy (SEM) analysis results shown, compared with the controls, QKI silencing increased the numbers of autophagosome-like vacuolar structures even without infection (Fig. 2H). When challenged with MRSA, more bacteria wrapped by autophagosome-like vacuolar in cytoplasm were observed in QKI silenced macrophages. Meanwhile, we also found that lysosomes were involved in antibacterial process of these two cells. Besides, to verify the effects of LC3 involved in autophagy in macrophages’ bactericidal activity, we used autophagy inhibitor, 3-MA (Fig. 2I, J) and Additional file 2: Figure S2E). As the results shown, increased expression LC3-II was confirmed after infection, but it was abolished by 3-MA. We also abolished the LC3-II expression by siRNA and found that the bactericidal ability of macrophages was suppressed in QKI deficiency cells (Fig. 2K, Additional file 2: Figure S2F and S2L). Moreover, the hallmark of autophagy, i.e., conversion of the LC3's soluble form to the LC3-II's (lipidated and autophagosome-associated form) was detected. Increased expressions of LC3-II, ATG5, and decreased expression of p62 were dramatically enhanced in the QKI deficient macrophages (Fig. 2L). These observations supported that autophagy enhancement facilitated the pathogen killing in QKI deficient macrophages.

### QKI loss mediated up-regulation of autophagy is dependent on PI3K-p110β

**Fig. 3:**
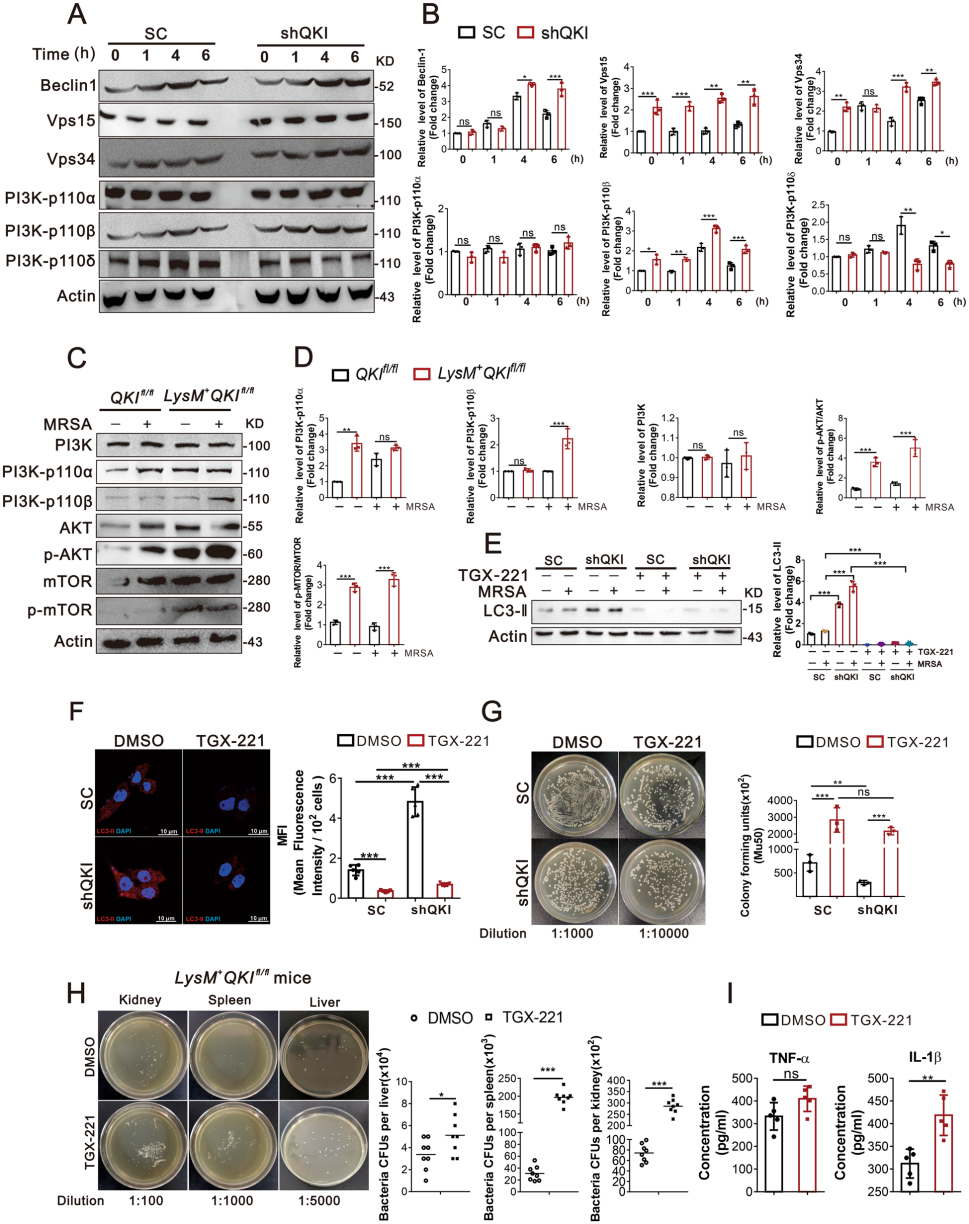
QKI loss mediated up-regulation of autophagy is dependent on PI3K-p110β. **A**, **B** Immunoblot analysis of protein expression of Vps15, Vps34, Beclin-1, PI3Kp110α, PI3K-p110β and PI3Kp110δ after SC and shQKI5 RAW264.7 cells infected with MRSA for indicated durations. Actin was an internal control. One representative immunoblot is shown (**A**) and the graph (**B**) presents quantification of the band intensity for immunoblots from three independent experiments. **C**–**D** Immunoblot analysis of PI3Kp110α, PI3K-p110β, PI3K, AKT, phospho-AKT (Ser473), mTOR, and phospho-mTOR (Ser2448) protein levels in lysates of bone marrow-derived macrophages (BMDM) from QKI^fl/fl^ and LysM^+^QKI^fl/fl^ mice stimulated with MRSA infection. Actin was an internal control. One representative immunoblot is shown (**C**) and the graph (**D**) presents quantification of the band intensity for immunoblots from three independent experiments. **E** Western blots were performed to determine the effects of inhibitor of PI3K-p110β “TGX-221” after MRSA infection 4 h. Total cell lysates were subjected to western blot to analyze the protein expression of LC3-II, with actin as an internal control. One representative immunoblot is shown (**E**). Graph (**F**) are representative quantification of the band intensity for immunoblots from three independent experiments. The results were shown as expression of LC3-II. **F** Immunofluorescence showed the expression of LC3-II in QKI silenced (ShQKI) and scramble (SC) RAW264.7 cells treated with TGX-221 under infection conditions. LC3-II was labeled in red and DAPI staining was shown in blue (Scale Bar: 10 μm). The mean fluorescence intensity of LC3-II immunostaining was showed on the right. **G** “Phagocytosis assay” was performed to determine the effect of TGX-221.The number of bacteria units forming were calculated after 8 h. Enumeration of forming bacteria units (c.f.u) were shown (right). Representative graph of bacteria forming units were showed in the left. **H** Representative imagines showed the bacteria forming units after LysM^+^QKI^fl/fl^ mice were treated with inhibitor of PI3K-p110β “TGX-221”. Enumeration of forming bacteria units (c.f.u) from various organs (kidney, spleen, liver) were analyzed **I** Cytokines (TNF-α and IL-1β) level in blood were analyzed by ELISA method after LysM^+^QKI^fl/fl^ mice were infected 24 h and treated with inhibitor of PI3K-p110β “TGX-221”. All the bars represented the mean of measurements from three independent experiments, and the error bars indicated ± SD. (A-E) are representative of three experiments. *p < 0.05, **p < 0.01, ***p < 0.001 (student’s t test). **H**, one-way ANOVA with Tukey’s multiple comparisons test. **G** are representative of one experiment (n = 3 wells/group). **H**, **I** are from one experiment (**H**, n = 8 mice/group I, n = 4 mice/group). Symbols represent data points for each animal. *p < 0.05, **p < 0.01, ***p < 0.001, not significant (ns) (student’s t test)

The mechanisms underlying QKI mediated autophagy were subsequently characterized. As mentioned earlier, PI3Ks is one of the key regulators in autophagy initiation, but PI3K-p110β does not promote autophagy by affecting the Akt-TOR pathway. Rather, it associates with the autophagy-promoting Vps34-Vps15-Beclin1-Atg14L complex and facilitates the generation of cellular PtdIns(3)P(24). QKI deletion significantly increased the protein levels of PI3K-p110β, Vps15, Vps34, in RAW264.7 cells (Fig. 3A and B) as well as bone marrow-derived macrophages (BMDM) with post-MRSA infection (Fig. 3C and D). As a contrast, the expression levels of PI3K-p110α, PI3K-p110δ remained unaltered compared to the control. The elevation of Akt-mTOR pathway in BMDM cells of QKI deficiency macrophages was also worthy to note (Fig. 3C). As stated by previous studies, PI3K-p110β, unlike other two members, positively induced autophagy. Thus, TGX-221, the inhibitor of PI3K-p110β was used to suppress autophagy. Notably, TGX-221 abrogated the increased autophagic activity of both control and QKI deficient cells (Fig. 3E). This finding was further validated using confocal imaging, TGX-221 mitigated the LC3-II expression post-MRSA infection (Fig. 3F). These data indicated that pathogen engulfment and clearance by macrophages via xenophagy are primarily mediated by a mTOR independent pathway. More importantly, it’s worthy to determine the role of PI3K-p110β in clearing bacterial remnants in the in vivo and in vitro models. TGX-221 treatment indeed completely blocked the bactericidal activity of macrophages post-MRSA infection in RAW 264.7 cells (Fig. 3G). Worthy to note that, TGX-221 treatment also abrogated the anti-bacterial activity of macrophages in LysM^+^QKI^fl/fl^ mice as demonstrated by an increased number of bacterial colonies in multiple organs, such as liver, spleen, and kidney (Fig. 3H). And the level of pro-inflammatory cytokines including (TNF-α and IL-1β) were further elevated in LysM^+^QKI^fl/fl^ mice after TGX-221 treatment in MRSA induced sepsis (Fig. 3I). These data validated that pathogen removal in macrophage is dependent on PI3K-p110β autophagy pathway. It’s intriguing to understand the underlying molecular mechanisms.

### PI3K-p110βmRNA, a direct mRNA target of QKI, was dynamically regulated by QKI in the cytoplasmic P-bodies

**Fig. 4:**
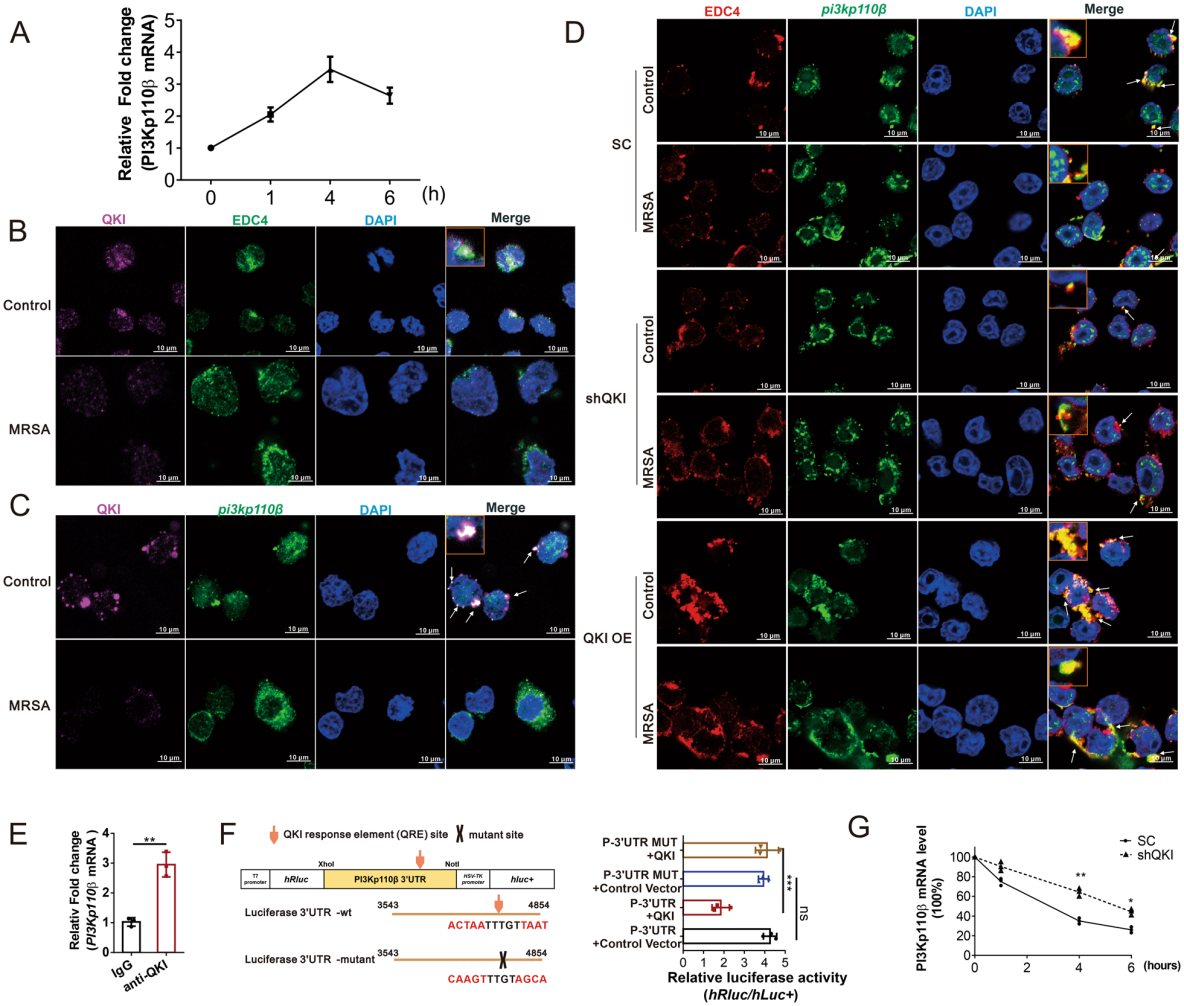
PI3K-p110β mRNA, a direct mRNA target of QKI, was dynamically regulated by QKI in the cytoplasmic P-bodies. **A** PI3K-p110β mRNA were analyzed after RAW264.7 cells incubated with MRSA for indicated time. **B** RAW264.7 cells transfected with plasmids encoding Flag-tagged QKI were fixed and doubly immuno-stained using anti-flag and anti-EDC4 antibody. Immunofluorescence showed the expression of flag-tagged QKI and EDC4, and DAPI. DAPI: blue, QKI: pink, EDC4: green (Scale Bar: 10 μm). The co-localization of QKI and EDC4 was showed on the top. **C** RNA-FISH detection of PI3K-p110β mRNA and immuno-stained with Flag-tagged QKI expressed RAW264.7 cells. Representative fluorescence images of protein expression of flag-QKI and localization of PI3K-p110β mRNA. DAPI:blue, PI3K-p110β mRNA:green, flag-QKI:pink (Scale Bar:10 μm). The co-localization of QKI and PI3K-p110β mRNA was showed on the top. **D** RNA-FISH detection of PI3K-p110β mRNA and immunostained with EDC4 in various cells. Representative fluorescence images of protein expression of EDC4 and localization of PI3K-p110β mRNA. DAPI:blue, PI3K-p110β mRNA:green, EDC4:red (Scale Bar:10 μm).The co-localization of EDC4 and PI3K-p110β mRNA was showed on the top. **E** RNA immunoprecipitation (RIP) was used in overexpress QKI RAW264.7 cells and the results indicated QKI protein interacts with PI3K-p110β mRNA. **F** Construction of luciferase reporter vectors (PI3K-p110β full-length 3’UTR), and PI3K-p110β 3’UTR QRE motif site mutant. Luciferase activity assays was used to examine the functional QRE motif site in PI3K-p110β 3’UTR affected by QKI. **G** The mRNA stability in macrophage was measured by incubating cells with actinomycin **D** to block transcription at the indicated time points. Real-time PCR was performed for the analysis of PI3K-p110β mRNA expression. All the bars represented the mean of measurements from three independent experiments, and the error bars indicated ± SD. (**B**–**D**) are representative of three experiments, **p < 0.01. (student’s t test). (**F**) are representative of three experiments. ***p < 0.001 one-way ANOVA with Tukey’s multiple comparisons test. (**G**) are representative of one experiment (n = 3 wells/group), *p < 0.05, **p < 0.01, not significant (ns). (student’s t test)

P-body is a typical cellular component encompassing a myriad of mRNAs and RNA metabolism related proteins. The mRNAs in P-bodies are either sequestered or stored, but not actively translated [14, 15]. Intriguingly we firstly found that mRNA level of PI3K-p110β was surged in first four hours after bacterial infection, but the protein level of PI3K-p110β was unchanged (Figs. 4A and 3C), suggesting that translation of its mRNA was depressed. Then we observed the dot-like staining of QKI in the cytoplasm, there’re co-localizations of QKI and EDC4 (P-body biomarker) in the cytoplasm of RAW264.7 cells, and the QKI was dramatically decreased after infection (Fig. 4B). It indicated that QKI may trap the target mRNAs in the P-body to repress its translation. This result was confirmed in mass spectrometry analysis, showing that P body specific proteins which were also detected in the QKI interacting protein complex, such as EDC3, EDC4, DDX6. The combination of QKI and EDC3 or DDX6 were verified by co-immunoprecipitation experiment (Additional file 2: Figure S2H).

Next, co-localizations of EDC4, QKI, and FAM-labelled PI3K-p110β mRNA probe were further observed by using the RNA-FISH assay. At rest state, QKI and EDC4 were colocalized with PI3K-p110β mRNA in the cytoplasm of uninfected cells displaying an ubiquitous dot-like staining patterns (Fig. 4C and D). However, the expression of QKI was decreased in post-MRSA infection, PI3K-p110β mRNA, was abruptly released from P-bodies, displaying a higher intensity level of FAM-labeled PI3K-p110β mRNA in the cytoplasm with an eccentric distribution. On the contrary, an augmented co-localization of EDC4 and PI3K-p110β mRNA was observed in macrophages after QKI overexpression (Fig. 4D). RIP (RNA Binding Protein Immunoprecipitation) analysis validated the direct interaction between QKI and PI3K-p110β mRNA (Fig. 4E). Bioinformatics analysis predicted one dual QRE site in the 3’UTR of PI3K-p110β mRNA (Fig. 4F). To further investigate it, a dual-luciferase reporter assay was constructed. As per the outcome of this analysis, QRE cis-element in suppressed the PI3K-p110β expression (Fig. 4F). Finally, we investigated the influence of QKI on PI3K-p110β mRNA stability. A significant delay in the PI3K-p110β mRNA degradation was observed in the QKI silenced cells (Fig. 4G). This, along with RNA-FISH data, validated QKI-mediated sequestration of PI3K-p110β mRNA in the P-body granules is targeted either for degradation or for temporary storage. We speculate that QKI reduction releases its trapping on PI3K-p110β mRNA in P-bodies, PI3K-p110β mRNA is released to the ER-ribosome regions for active translation. This is the first time to disclose QKI was mainly present in the P body region of macrophages at rest state and QKI reduction facilitated the target mRNA transporting to the translational machine to enhance Xenophagy process in post-infection.

### MRSA infection induced decreased QKI expression was mediated by the ubiquitin–proteasome system

**Fig. 5:**
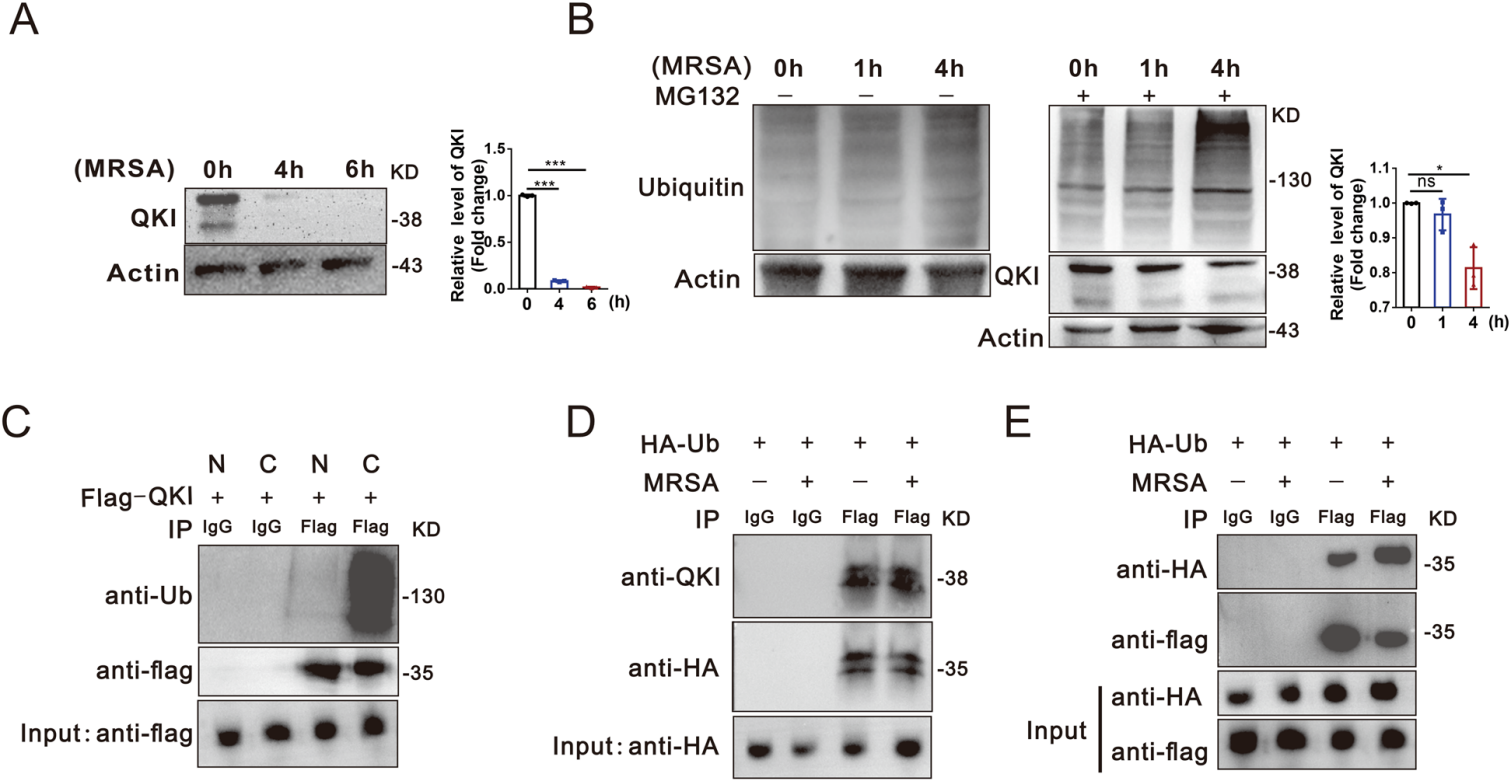
Macrophage QKI depletion in post-MRSA infection was mediated by the ubiquitin–proteasome system. **A** Protein level of QKI was determined by western blot after RAW264.7 cells were stimulated by MRSA for indicated time. One representative immunoblot is shown (left). Graph (right) are representative quantification of the band intensity for immunoblots from three independent experiments. **B** Immunoblot analysis of whole-cell lysates from Raw264.7 cells after treatment of inhibitor “MG132” and infection with Mu50 for indicated durations. One representative immunoblot is shown(left), Graph (right) are representative quantification of the band intensity for immunoblots from three independent experiments. **C** RAW264.7 cells were incubated with bacteria and ubiquitin inhibitor “MG132” for 4 h, followed by nuclear (N) and cytoplasmic protein (C) extraction. Then the lysates were subjected to immunoprecipitation using anti-Flag and immunoblotted with indicated antibodies. **D** Immunoblot analysis of immunoprecipitated HA-tagged ubiquitin transiently expressed in RAW264.7 cells and incubating with Mu50 for 4 h, Ub, ubiquitin. The whole-cell lysates were subjected to immunoprecipitation using anti-HA under denaturing conditions, and immunoblotted with indicated antibodies. (**E**) RAW264.7 cells were co-transfected with indicated plasmids. At 36 h post-transfection, cells were infected for 4 h and lysed, followed by immunoprecipitation using anti-flag under denaturing conditions, followed by immunoblotting with indicated antibodies. All the bars represented the mean of measurements from three independent experiments, and the error bars indicated ± SD. (**A**–**E**) are representative of three experiments, **A**, ***p < 0.001 (student’s t test). **B**, *p < 0.05, not significant (ns), one-way ANOVA with Tukey’s multiple comparisons test

To assess the regulation and biological significance of QKI expression during MRSA-induced sepsis, the expression level of QKI was determined in the RAW264.7 cells post-MRSA infection. We observed that the QKI protein level was reduced significantly at 4 h and undetectable at 6 h post-MRSA infection (Fig. 5A). The prompt reduction of QKI led us to speculate that protein degradation by ubiquitination related proteosome are more likely. The ubiquitin inhibitor, MG132, restored the QKI expression significantly, meanwhile increased total levels of ubiquitinated proteins, which validated our hypothesis (Fig. 5B). Moreover, ubiquitinated QKI was primarily in the cytoplasm of RAW264.7 cells after infection (Fig. 5C). The QKI ubiquitination was confirmed by co-immunoprecipitation experiment (Fig. 5D and E). These data indicated that ubiquitin–proteasome participated in the QKI degradation in macrophages post-MRSA infection.

### Ubiquitination of QKI was mediated by RNF6

**Fig. 6:**
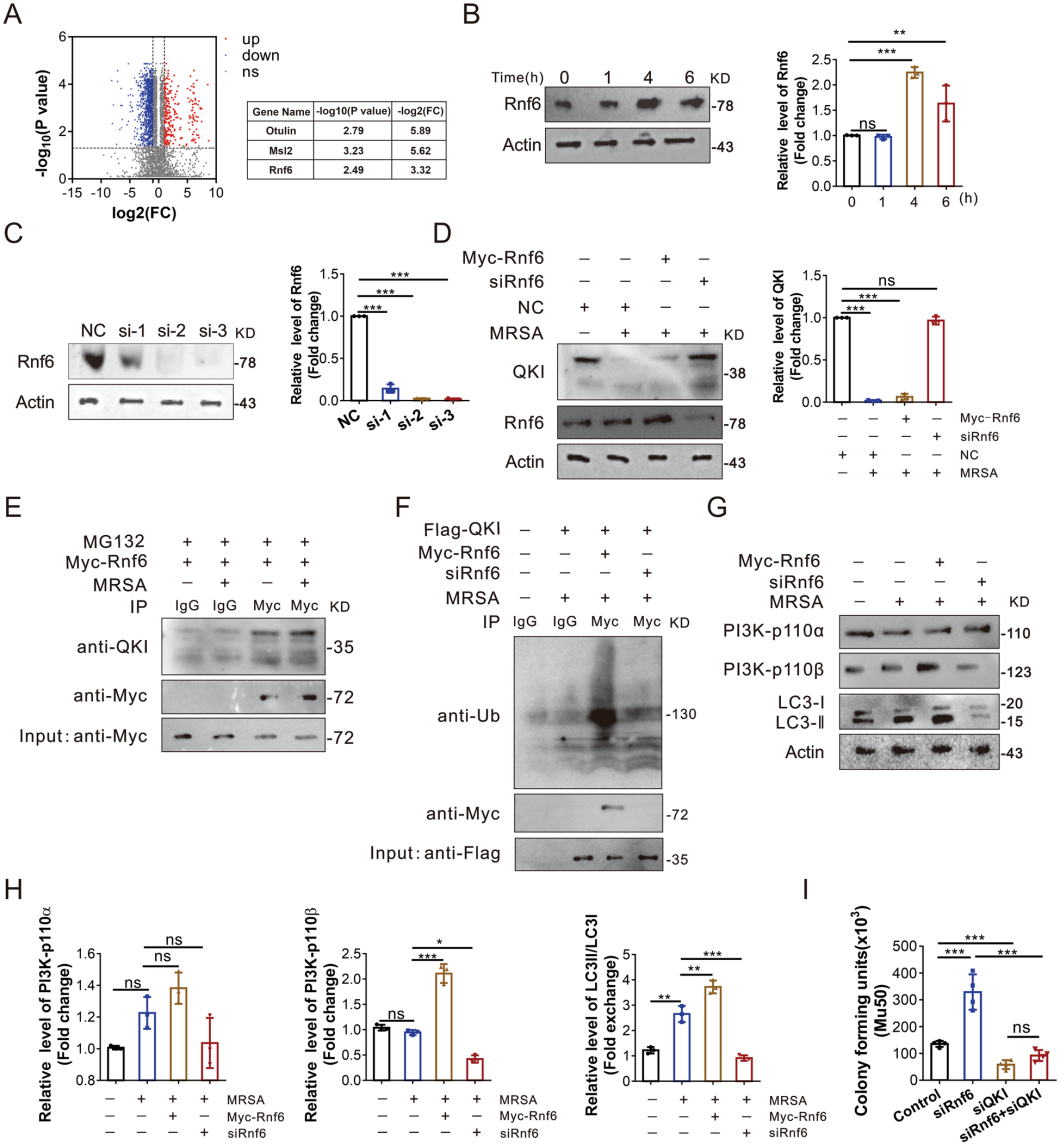
Ubiquitination of QKI was mediated by RNF6. **A** Identification of differentially expressed proteins between flag-QKI –transfected (infection) and flag-QKI control (without infection)– transfected RAW264.7 cells. Negative controls with or without infection were internal controls respectively. LC–MS/MS, liquid chromatography coupled to tandem mass spectrometry. Volcano graph showed different expressions of protein after infection. The y-axis corresponds to the mean expression value of log10 (p-value), and the x-axis displays the log2 fold exchange value. The red dots represent the up regulated expressed transcripts [(p < 0.05) and fold change > 2]. The blue dots represent the transcripts whose expression down regulated [(p < 0.05) and fold change > 2]. Red and blue dots represent probe sets for protein expression at significantly higher (n = 70) or lower (n = 59) levels during infection. **B** Immunoblot analysis of expression of Rnf6 after RAW 264.7 cells infection for indicated durations, with actin as an internal control. One representative immunoblot is shown (on the left). Graphs (on the right) are representative quantification of the band intensity for immunoblots from three independent experiments. **C** RAW264.7 cells were infected with siRNA for Rnf6 (Si-1, Si-2, Si-3) and NC for control. Cell lysates were subjected to western blot to analyze the protein expression of Rnf6, with actin as an internal control. One representative immunoblot is shown (on the left). Graphs (on the right) are representative quantification of the band intensity for immunoblots from three independent experiments. **D** Immunoblot analysis of expression of QKI and Rnf6 after Raw264.7 was transfected with Myc-tagged Rnf6 vector, siRnf6, and NC vector with Mu50 infection or not. One representative immunoblot is shown (on the left). Graphs (on the right) are representative quantification of the band intensity for immunoblots from three independent experiments. **E** Immunoblot analysis of immunoprecipitated Myc-tagged Rnf6 transiently expressed in RAW264.7 cells. After infection of Mu50 for 4 h, the whole-cell lysates were subjected to immunoprecipitation using anti-Myc under denaturing conditions, and immunoblotted with indicated antibodies. **F** RAW264.7 cells were transfected with Myc-tagged Rnf6 vector, Flag-tagged QKI vector and siRNA of Rnf6 as indicated. After infection of Mu50 for 4 h, the whole-cell lysates were subjected to immunoprecipitation using anti-Flag under denaturing conditions, and immunoblotted with indicated antibodies. **G**–**H** Immunoblot analyzed the expression of PI3K-p110α, PI3K-p110βand LC3 in RAW264.7 cells after transfected with Myc-tagged Rnf6 and siRnf6. After infection of Mu50 for 4 h, the whole-cell lysates were subjected to immunoblot analysis using indicated antibodies. One representative immunoblot is shown. Graphs (Figure H) are representative quantification of the band intensity for immunoblots from three independent experiments. **I** RAW264.7 cells were transfected with siRNA of siRnf6, siQKI or both respectively, followed with phagocytosis assay. At 8 h post-incubation, the number of bacteria units forming was counted. Enumeration of forming bacteria units (c.f.u) were showed. All the bars represented the mean of measurements from three independent experiments, and the error bars indicated ± SD. (**B**–**H**) are representative of three experiments, *p < 0.05, **p < 0.01, ***p < 0.001, not significant (ns), one-way ANOVA with Tukey’s multiple comparisons test

In order to identify the interacting E3 ubiquitin proteins tagged for QKI in macrophage after MRSA-infection, the co-immunoprecipitation analysis was performed. Among all QKI interacting proteins identified by mass spectrometry, three E3 ubiquitin related candidate proteins were selected (Fig. 6A). Among these proteins, only siRNA specific to RNF6 could increase QKI protein level (Fig. 6C and D, Additional file 2: Figure S2F and S2G). Moreover, the RNF6 level was subsequently increased post-MRSA infection, being negatively correlated to the QKI expression (Fig. 6B). The co-immunoprecipitation analysis showed that Rnf6 could interact with QKI in macrophages regardless of infection (Fig. 6E). Ectopic expression of RNF6 increased the level of ubiquitinated QKI, while RNF6 knockdown cells failed to exhibit similar effect (Fig. 6F). Accordingly, ectopic expression of Rnf6 also enhanced the levels of PI3K-p110β and ratio of LC3-II /LC3-I which indicates enhanced autophagy, while siRNA specific to RNF6 displayed the reverse effects. Taken together, the up-regulated Rnf6 post-infection promoted QKI degradation and facilitated the release of PI3K-p110β mRNA from P body to activate translation, ending up with its higher expression and the enhanced autophagy (Fig. 6G and H). At last, we verify the effect of Rnf6 in antibacterial process in macrophages. As the data shown (Fig. 6I and Additional file 2: Figure S2J), the bactericidal ability was dramatically dampened after knockdown siRnf6 of macrophages, but it was restored after silencing QKI by siRNA.

### Nanoparticles containing siRNA targeting QKI improved the survival rate of MRSA-infected mice

**Fig. 7:**
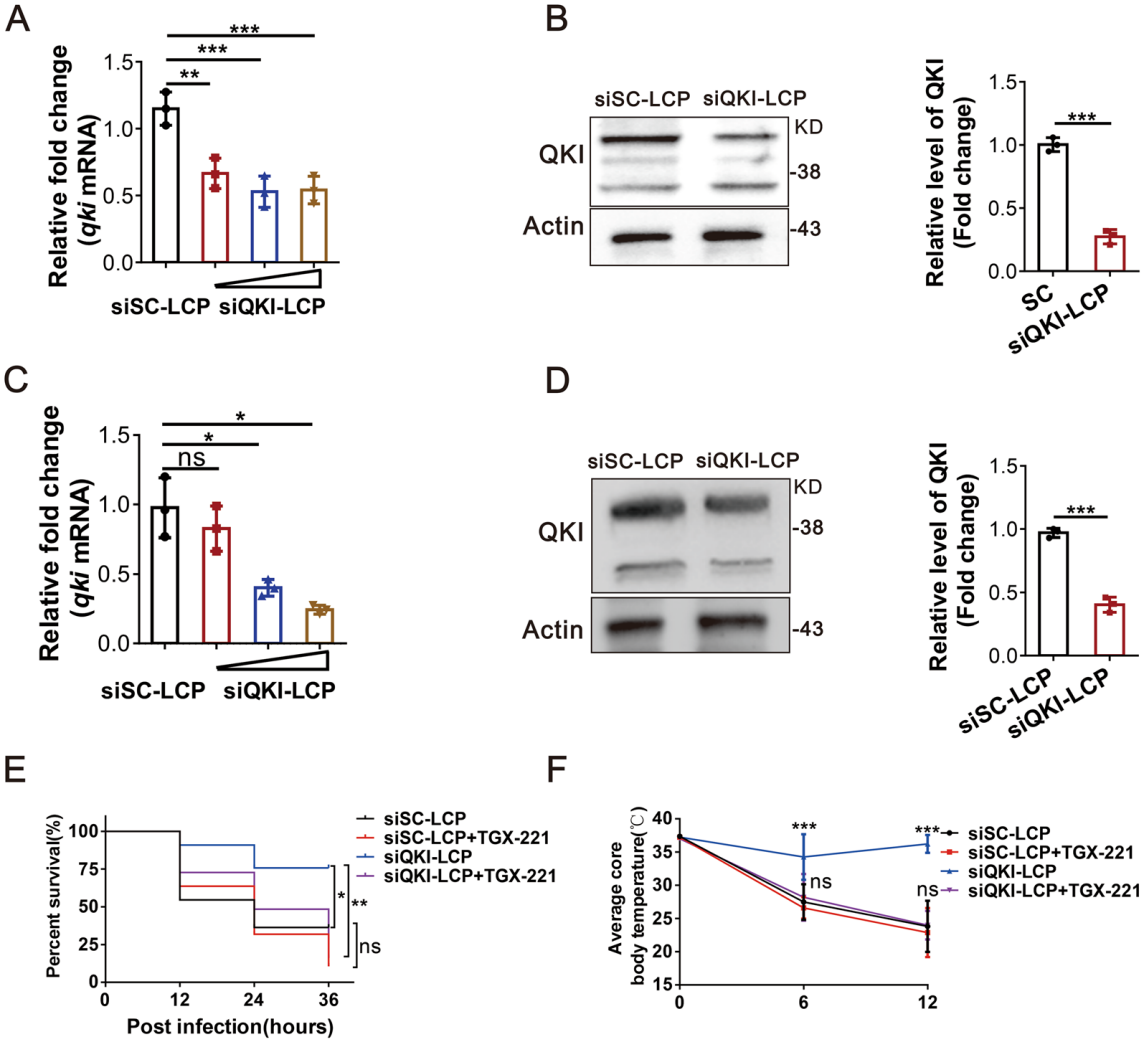
Nanoparticles containing siRNA targeting QKI improved the survival rate of MRSA-infected mice. **A** Real-time PCR analysis of relative mRNA expression of QKI in RAW264.7 cells after treated with siQKI-LCP in various dose. **B** Western blots were performed to determine the effects of siQKI-LCP incubated with RAW264.7 cells 24 h before MRSA infection. Total cell lysates were subjected to western blot to analyze the protein expression of QKI, with actin as an internal control. One representative immunoblot is shown (on the left). Graphs (on the right) are representative quantification of the band intensity for immunoblots from three independent experiments. **C**, **D** C57/B6 mice were intraperitoneally injected with siQKI-LCP followed with MRSA (1 × 10^9^) infection in the next day. **c** Real-time PCR analysis of relative mRNA expression of QKI in monocytes purified from peritoneal cells. **d** Western blots were performed to determine the effects of siQKI-LCP in purified monocytes after MRSA infection 24 h. Total cell lysates were subjected to western blot to analyze the protein expression of QKI, with actin as an internal control. One representative immunoblot is shown (on the left). Graphs (on the right) are representative quantification of the band intensity for immunoblots from three independent experiments. **E**, **F** C57/B6 mice were intraperitoneally injected with siQKI-LCP, siSC-LCP, TGX-221(3 mg/kg) respectively followed with MRSA (1 × 10^9^) infection in the next day. (e) The survival rate of mice infected with MRSA within 3 days. (f) The body temperature of mice within 12 h was measured (n = 12/group). All the bars represented the mean of measurements from three independent experiments, and the error bars indicated ± SD. **A**, **C** are representative of three experiments, **p < 0.01, ***p < 0.001, one-way ANOVA with Tukey’s multiple comparisons test. **B**, **D** are representative of three experiments, ***p < 0.001(student’s t test). (**E**, **F**) are representative of one experiment (n = 12 mice/group). **E**, *p < 0.05 Log-rank (Mantel-Cox) test; **F**, ***p < 0.001, (student’s t test)

To further explore the therapeutic role of QKI in sepsis with pathogen infection, we constructed lipid-coated particles encapsulated with QKI specific siRNA, termed as “siQKI-LCP”. Then, siQKI-LCP was successfully transfected into peritoneal macrophages and RAW264.7 cells at different concentrations and the mRNA and protein levels of QKI were decreased in peritoneal macrophages (Fig. 7A and B) and RAW264.7 cells (Fig. 7C and D). C57/BL6 mice infected with MRSA were intraperitoneally injected with either siQKI-LCP or siSC-LCP. We observed that siQKI-LCP treatment significantly improved the survival rate and body temperature of MRSA-infected mice, but the inhibitor of PI3K-p110β, TGX-221 abolish the effect of both groups. (Fig. 7E and F).

## Discussion

Microbial infection is a major cause of nosocomial sepsis or community-acquired sepsis. Administration of appropriate antibiotics at an early stage of sepsis is the primary option for treatment. However, with the extensive use of antibiotics, antibiotic-resistant bacteria have emerged as a global health problem over the time. Among these drug-resistant bacteria, methicillin-resistant *S. aureus* (MRSA) causes the most severe infection with a high fatality rate [24]. Therefore, searching for novel therapeutic strategies beyond current antibiotic treatment is urgently needed.

In response to microbial invasion, phagocytes are rapidly recruited to sites of injury to eliminate pathogens via phagocytosis and secretion of anti-bacterial molecules, such as nitric oxide (NO) and ROS, and pro-inflammatory cytokines including tumor necrosis factor-α (TNF-α), IL-1β, and IL-6 [25]. In this study, as our findings, LysM^+^QKI^fl/fl^ mice displayed higher survival rate and lower bacterial burden as well as elevated levels of pro-inflammatory cytokines compared to the littermates both in MRSA and CLP induced septic models. In addition, in vitro investigations also validated the fact that QKI deletion enhanced the phagocytic and bactericidal activity in macrophages (Additional file 1: Figure S1A–E). In contrast to the protection effects in microbial infection, QKI deletion was harmful in septic model challenged with LPS, which was reported in our previous study [20]. The totally inverse influences of QKI on these two different septic models were similar with kruppel-like transcription factor 2 (KLF2) [26]. Consisted with our results, Jain et al.also observed that LPS-induced model displayed significantly higher levels of proinflammatory cytokines than that in the CLP models. Therefore, these results strongly suggest that increased inflammation led by QKI deletion is good to pathogen clearing but bad to LPS challenge, thus leading to different effects in two septic models. Moreover, we noticed a significantly reduced level of QKI in monocytes and PBMCs of whole blood collected from septic patients and QKI mRNA level decreased as sepsis progressed. These data indicated that the changes in macrophage QKI expression was identical in sepsis patients.

Autophagy is one of the essential processes of innate immunity against invading pathogens. In this process, the invading microbe is internalized by macrophages, sequestrated in autophagosomes, and transported to the lysosomes for degradation [11, 27, 28]. Our observation disclosed the co-localizations of autophagic vacuoles with FITC-Mu50 in parallel with the higher autophagic indicators, including ATG5 and LC3-II/LC3-I ratio, but the lower P62. Moreover, autophagy inhibitor ablated the pathogen removal effects caused by QKI deletion in macrophages. In terms of autophagy, the initiation and progression were finely controlled. One of the pivotal groups of kinase controlling the autophagy is the phosphatidylinositol 3-kinases (PtdIns3Ks) and the phosphoinositide3-kinases (PI3Ks). Out of the three classes of PI3Ks, class I PI3K at the plasma membrane suppresses autophagy through activating Akt/mTOR complex 1 pathway. Class IA PI3Ks are heterodimeric proteins consisting of an 85 kDa regulatory subunit and one 110 KD catalytic subunits out of three subunits, i.e., p110-α, p110-β, and p110-δ [12]. Opposite with the anti-autophagic role of either p110-α or p110-δ, p110-β as a growth factor limited sensor, acts as scaffold protein to promote autophagy via forming Vps34-Vps15-Beclin 1-Atg14L complex and the production of PtdIns(3) [29, 30].

Here, both in vitro and in vivo models challenged by MRSA, QKI deficiency led to higher levels of PI3K-p110β, Vps34 and Belin1 in macrophages. However, TGX-221, a PI3K-p110β inhibitor, abolished the effects of QKI deficiency on autophagy and bactericidal activity in macrophages. These evidences strongly supported that QKI deficiency promoted autophagy via PI3K-p110β dependent pathway.

Worthy to note that the levels of AKT and mTOR are increased in QKI deficiency macrophage after infection, it indicated that the increased pathogen removal effects were independent on the classical mTOR pathway. Moreover, the QKI deficit cells confer the higher survival ability. That may explain the protective effects related with pathogen infection in QKI deficit mice.

As bioinformatics analysis indicated that PI3K-p110β mRNA contains one QRE site in the 3′UTR region and is a putative direct target of QKI. RIP and dual-luciferase reporter assay validated the prediction. Herein, we firstly observed that QKI and PI3K-p110β mRNA were co-localized with the P body marker, the enhancer of decapping 4 (EDC4) protein. Persistent QKI expression sequestered PI3K-p110β mRNA in P body. QKI reduction by shRNA or infection facilitated the release of PI3K-p110β mRNA from P body. Since P body is regarded as a storing site for mRNA more recently, our data provided the direct evidences to support that some specific mRNA, like PI3K-p110β, are trapped by the RNA-binding protein, QKI in P body at rest state. Stress signal, such as infection, promptly discharged the untranslated mRNA stores away from the P body to achieve the active protein translation. QKI, as a RNA-binding protein is firstly reported to be localized in P body.

In order to define the mechanisms of decreased protein level, QKI-IP complex was analyzed by proteomic mass spectrometry. Among various positive interacting proteins, Ring finger protein 6 (RNF6) was obtained. RNF6, a RING-type E3 ubiquitin-protein ligase, acts as an oncogene in multiple cancers [31, 32]. RNF6 degrades its target protein in a ubiquitin-dependent manner. However, the functional role of RNF6 and its target protein in sepsis remains elusive so far. RNF6 level was significantly increased in macrophages post-infection. Moreover, in vitro experiments also validated that RNF6 mediated ubiquitinated QKI for degradation. Finally, RNF6-QKI-PI3K-p110β-autophagy pathway are contributing to the enhanced bacterial killings in macrophage. Besides there’re other P body specific proteins which are also detected in the QKI interacting protein complex in the mass spectrometry analysis, such as EDC3, EDC4, DDX6 (Additional file 2: Figure S2H). Further detailed characterizations are still required in future.

What is more, in order to determine the anti-bacterial effect of QKI, we constructed the nanoparticle containing QKI-specific siRNA termed as siQKI-LCP. The in vivo results supported the effectiveness of siQKI-LCP in sepsis treatment.

As with majority studies, there are some limitations in the present study that could be addressed in future research. First, recent studies revealed that a non-canonical function of autophagy proteins, LC3-associated phagocytosis (LAP), which functions in engulfing particles, including pathogens, and dying cells, thus modulating macrophage immune responses. Although both the LAP pathway and that of canonical autophagy share several key molecular regulators, they exert some distinct proteins. Autophagy-related proteins including ULK1/2, ATG13, FIP200, and ATG14 are dispensable for LAP [33], while LAP-specific proteins (dispensable for autophagy) are Rubicon (RUBCN) or NADPH oxidase 2 (NOX2) [34, 35]. In this study, we observed the expression changes of VPS34, VPS15, BECN1, ATG5, and LC3 in QKI deficient macrophages, all of which are shared by LAP and canonical autophagy pathway [36]. Second, to prove that QKI deletion increased the bactericidal activity of macrophages by promoting autophagy, 3MA (a Vps34 inhibitor) was used. However, 3MA is not an autophagy specific inhibitor, which also inhibits the non-autophagy related PI3KC3 functions, such as LAP. Third, LC3II is essential for the execution of autophagy and therefore is the widely accepted marker for autophagy activity assessment. As it is well known, there are at least 7 Atg8 orthologs in mammals, which display 92, 94, 71, 59, 60, and 65% sequence similarity, respectively, in comparison with LC3II. Nevertheless, we synthesized a siRNA specific for LC3 II reported by previous study [37] and verified its effects on knocking down LC3 II expression by western-blot analysis (shown in Additional file 2: Figure S2L) but not on other homologues. The above limitations indicated that the involvement of LAP may not be ruled out. Therefore, to explore the roles of LAP and canonical autophagy in the protection effects induced by QKI knock-down, SBI-0206965 (an ULK1 inhibitor, inhibit autophagy but not LAP), and GSK2795039 (a NOX2 inhibitor, suppress LAP but not autophagy) were used, and the anti-bacterial effects in QKI deficient macrophages were analyzed. As we expected, SBI-0206965 suppressed the bactericidal activities in QKI ablated macrophages (shown in Additional file 2: Figure S2M), which further supported the conclusion in this study. Interestingly, addition of GSK2795039 also displayed similar effects (shown in Additional file 2: Figure S2M). This finding suggested that besides canonical autophagy, LAP might also contribute to the increased bactericidal ability induced by QKI ablation. However, to prove this, more works are needed. For example, we need to establish mice model not only with conditional ablation of QKI, but also with myeloid cells deficient in FIP200, ULK1, or ATG14 lacking autophagy but LAP is not influenced, while such mice deficient for Rubicon (RUBCN) or NADPH oxidase 2 (NOX2) lack LAP but not autophagy. Then these mice were challenged with MRSA and the corresponding indicators were observed, which will be done in our future work.

## Conclusions

In summary, we demonstrated QKI ablation in macrophages was protective in MRSA induced sepsis model. QKI deletion increased the bactericidal activity of macrophages by promoting autophagy through PI3K-p110βactivation. We identified the interaction of RNF6 with QKI via ubiquitin mediated degradation and facilitated the escape of PI3K-p110βmRNA from P body for active translation. Therefore, therapeutic intervention of QKI might be a novel strategy for the treatment of poly-microbial especially MRSA induced sepsis.

## Methods

### Cells

RAW 264.7 cells (American Type Culture Collection) were cultured in DMEM with 10% fetal bovine serum (Gibco by Life Technologies). QKI silencing and overexpression in RAW264.7 cells were achieved as described before [22]. Here, cells were represented as SC (Scramble), shQKI and QKI. Mouse peritoneal macrophages were obtained as described previously [22]. Three days before collecting peritoneal cells, 6- to 8-week-old mice were injected with 1 ml of 3% thioglycolate medium. Cells were further enriched by discarding the culture medium containing non adhesive cells after seeding into 6-well plates for 2 h. Bone marrow-derived macrophages (BMDM) were harvested as previously described[22]. In brief, bone marrow cells were collected from femur of male QKI^flox/flox^ and QKI^flox/flox^-CreLySM mice. Then these cells were seeded into plastic petri dish in complete RPMI 1640 medium supplemented with 20 ng/ml recombinant murine macrophage colony stimulating factor (M-CSF, Peprotech, Rock Hill, USA) for 7 days. Following that, adherent cells were washed with PBS, and cellular infection was carried out through incubating with live *S. aureus* for indicated time.

### Cloning of RNF6 and DNA plasmids constructs

RT-PCR was used to isolate full-length cDNAs of Rnf6 encoding mouse on mRNA prepared from whole Raw264.7 cells, then cDNAs was subcloned into pCS2-MT vector with endonuclease sites of NotI and ApaI.

### siRNA transfection

RAW 264.7 cells were seeded in 6-well plates at a density of 7 × 10^5^ cells/well in antibiotic-free DMEM, and were transfected with 50 nM of siRNA (Genepharma, China) using the transfection reagent lipofectamine 2000 (Thermo Fisher, USA). After 48 h of transfection, the cells were infected with bacteria for indicated time. The cells were then used for the Western blot and the supernatants were collected for phagocytosis analysis. The siRNA sequences were as follows:

siQKI 5′-GAACAAAGAAACCCUUUAUTT-3′ (forward) and 5′-AUAAAGGGUUUCUUUGUUCTT-3′ (reverse); siLC-3 5′-GCUCCUGAUCUGCUAAUAATT-3′ (forward) and UUAUUAGCAGAUCAGGAGCTT-3′ (reverse); siRnf6-1 5′- GCUAAUGAGAGACCAUAAUTT-3′ (forward) and AUUAUGGUCUCUCAUUAGCTT-3′ (reverse); siRnf6-2 5′- GCAAAUAGAACCCGAUCUATT-3′ (forward) and UAGAUCGGGUUCUAUUUGCTT-3′ (reverse); siOtulin 5′- GGAUAUCAGAACCCAGGUUTT-3′ (forward) and AACCUGGGUUCUGAUAUCCTT-3′ (reverse); siMsl-1 5′- GCACUUCAUGGGUUAUCAUTT-3′ (forward) and AUGAUAACCCAUGAAGUGCTT-3′ (reverse); Negative control 5′-UUCUCCGAACGUGUCACGUTT-3′ (forward), 5′-ACGUGACACGUUCGGAGAATT-3′ (reverse);

### Mice

Male C57BL/6 mice [8–10 weeks old] were purchased from the Experimental Animal Center of Fourth Military Medical University. QKI conditional knock (LysM^+^QKI^fl/fl^) mice used were generated in the same way as Wang described [23]. In brief, heterozygous QKI-floxed transgenic (QKI fl/wt) mice were gener-ated. These mice were backcrossed with C57BL/6 mice (Animal Center of the Fourth Military Medical University) for 11 generations under specific, pathogen-free conditions. Heterozygous breeding pairs were used to generate homozygote QKI ^fl/fl^ mice. QKI conditional knock mice (LysMCre QKI^fl/fl^) were generated byserial breeding of QKI ^fl/fl^ mice with mice that have a Cre recombinase controlled by a LysM promoter, in which the conditional QKI allele is excised in myeloid cells. Age-matched QKI^fl/fl^ and LysMCre QKI^fl/fl^ (mice were heterozygous for LysM Cre) male mice were used in all experiments. All mouse experiments and procedures were approved by the Laboratory Animal Center of Fourth Military Medical University and conducted in conformity with the ethical standards. Mice were housed at the animal care facility at 22 °C with 12-h light/dark cycles. All mouse experiments and procedures were approved by the Laboratory Animal Center of Fourth Military Medical University.

### Bacterial strains and phage used

Methicillin-resistant *Staphylococcus aureus* (MRSA) strain Mu50 / ATCC 700699 was gift from Northwest A&F university. They were grown in LB media. The cultures were grown overnight, followed by subculture until logarithmic phase (A600 nm = 0.8).

### Induction sepsis model

C57BL/6 J male or transgenic mice [6–8 weeks] were used. Mice were injected intraperitoneally with live *S. aureus* / ATCC 700699 (1 × 10^9^ or 1.5 × 10^9^ CFU per mouse) or pyrogen-free phosphate-buffered saline (PBS) alone. Body temperature was measured using a rectal thermometer at various time points after infection. Lung, spleen, liver, and kidney were collected and homogenized at 24 h after infection. The homogenates were serial diluted in PBS and plated on tryptic soya agar plates. Plates were incubated at 37 °C overnight and the number of colonies were calculated. For survival studies, mice were observed for 3 days following infection. Death of mice was recorded and the data were analyzed for statistical significance of differences between groups.

### Real-time PCR

Total RNA was extracted with Trizol reagent (Life Technologies) and2μg of total RNA was reverse transcribed with M-MuLV reverse transcriptase (Takara) and mixture of random and oligo-dT primers.

Real-time PCR were performed with universal SYBR Green PCR Master mix on BioRad CFX96 Real-Time PCR Detection System (BioRad) with specific primers.

TLR2 5 ′-GCAAACGCTGTTCTGCTCAG-3′ (forward) and 5 ′-AGGCGTCTCCCTCTATTGTATT-3′ (reverse);

Dectin1 5 ′-GACTTCAGCACTCAAGACATCC-3′ (forward) and 5 ′-TTGTGTCGCCAAAATGCTAGG-3′ (reverse);

QKI (mouse) 5´-TAGCAGAGTACGGAAAGACATG-3´(forward) and 5´-GGGTATTCTTTTACAGGCACAT-3´(reverse);

β-actin (mouse) 5´-GTGACGTTGACATCCGTAAAGA-3´(forward) and

5´-GCCGGACTCATCGTACTCC-3´(reverse).

QKI (human) 5´-AGAGCAGTTGAAGAAGTGAAG-3´(forward) and 5´-AGAAGGTCATAGGTTAGTTGCC-3´(reverse).

β-actin (human) 5′- GGCTACAGCTTCACCACCAC -3′ (forward) and 5′- TGCGCTCAGGAGGAGC -3′ (reverse).

### Western blot

Protein lysates were harvested from macrophages using lysis buffer containing 50 mM Tris–Cl pH 7.4, 150 mM NaCl, 1% NP-40, 1 mM EDTA-free protease inhibitor cocktail (Roche) and 1 mM phenylmethylsulfonyl fluoride (PMSF). 50 μg of lysates were separated by SDS-PAGE and transferred to PVDF membrane (Millipore) for western blot analysis with specific primary antibodies (1:1000) for Ubiquitin (abcam, cat#ab13493), Msl2(CST,cat#ab44006), Outlin (abcam, cat# ab211328), p62 (Cell Signaling Technology, cat#ab16177), PI3Kp110α (Cell Signaling Technology,cat#ab4255), PI3K-p110β(Cell Signaling Technology,cat#ab3011), PI3Kp110δ(Cell Signaling Technology,cat#ab5405), Vps34(Cell Signaling Technology,cat#ab4263, AKT(Cell Signaling Technology,cat#ab4685), p-AKT(Ser473) (Cell Signaling Technology,cat#ab4060), mTOR(Cell Signaling Technology,cat#ab2972), Phospho-mTOR (Ser2448) (Cell Signaling Technology,cat#ab5563) and LC3 (Cell Signaling Technology, cat#ab4108), QKI (Sigma), LC3-II(Cell Signaling Technology, cat#ab2775), flag(Sangon, cat#D191041), HA(Sangon, cat#D191044), and Myc(Sangon, cat#D199941), Rnf6 (Thermo, cat#PA5-59044), EDC4 (proteintech, cat#D17737-1-AP) and β-actin(Sangon, cat#D191047). Immunolabelled proteins were detected by using appropriate HRP-conjugated secondary antibodies (Thermo, cat#31460), followed by visualization with ECL (Sangon).

### Immunoprecipitation assay

Cells were lysed in IP buffer (50 mM Tris–Cl, pH 7.5, 150 mM KCl, 0.5% NP40, 1 mM PMSF) at room temperature for 15 min with rotating. The lysate was centrifuged at 13,000 rpm for 10 min at 4 °C. The supernatant was collected and incubated with appropriate antibodies overnight at 4 °C. Then the mix of supernatant and antibodies was immobilized on Pierce A/G magnetic beads (Thermo) at 4 °C overnight with rotating. The beads were subsequently washed three times with IP buffer. For detection of polyUb chains on QKI, beads were washed with IP buffer supplemented with 0.1% SDS. The bound material was fractionated by SDS-PAGE followed by Western blot analysis.

### Protein extraction and iTRAQ mass spectrometry analysis

RAW264.7 cells were seeded in 10-cm dishes and transfected with Flag-QKI or Control vector for 36 h, followed by bacteria infection for 4 h in vitro. The analysis procedures in the same way as Zhao described [38]. The cells were harvested and lysed in complete lysis kit (Roche) with protease and phosphatase. Each sample (100 mg of protein) was digested with trypsin solution and labeled with the iTRAQ reagents (Applied Biosystems) with 113, 114, 115, or 116 reporter ions according tothe manufacturer’s protocol. Subsequently, the labeled peptides were mixed equally and separated by 1260 Infinity HPLC (Agilent Technologies), followed by nano liquid chromatography tandem mass spectrometry using the Hybrid Quadrupole-Orbitrap mass spectrometer (Q-Exactive; Thermo Fisher Scientific) equipped with a nano-UPLC RSLC Ultimate 3000 (Dionex). Both peptide identification and quantitation were performed in an overall workflow in Proteome Discoverer software (version 1.4; Thermo Fisher Scientific) and searched against the UniProt human canonical sequence protein database (October 7, 2011; 56,869 entries) using Mascot search engine (version 2.4). For protein identification, 95% confidence was used. For quantitation and further validation experiments, all reported data were based on 95% confidence for protein identify cation as determined by Proteome Discoverer (Unique peptide > 1).

### Dual-luciferase reporter assay

3′UTR of PI3K-p110β was introduced in to psiCHECKTM-2 reporter (Promega) with endonuclease sites NotI and XhoI, and complementary sequence mutation sites of seed sequence was designed on PI3K-p110β-3′UTR(P-3′UTR) to establish PI3K-p110β-mutant (P-3′UTR MUT). The dual luciferase reporter plasmids P-3′UTR and P-3′UTR were co-transfected with QKI or control vector respectively into RAW264.7 cells. Cells were harvested and lysed 48 h after transfection, and the dual-luciferase reporter assay kit (Beyotime Biotechnology Co., Ltd., Shanghai, China) was used to detected Renilla luciferase assay and firefly luciferase separately. The Renilla luciferase served as internal control. The transfection experiments were performed in triplicate for each plasmid construct.

### Phagocytosis assay

Raw264.7 cells or peritoneal cells were seeded into a 24-well plate (2 × 10^5^ cells/well) (Corning, USA) followed by co-cultured with MRSA at the ratio of 1:50 for 1 h. Then gentamicin (30 μg/mL, MedChem Express, USA) was added into wells to kill extracellular bacteria. For phagocytosis assay, cells were lysed and plated onto LB media. The number of bacteria colonies was counted after 12 h. For assessing the bactericidal ability of macrophages, after incubated with gentamicin, cells were cultured for another 8 h to allow intracellular bacteria killing. Then cells were lysed and plated onto LB media to count bacteria colonies 12 h later.

For detection of phagocytosis of RAW264.7 cells using Phagocytosis Assay Kit (IgG FITC) (NO.500290, USA), RAW264.7 cells (2 × 10^5^cells/ml) plated on a 4 well chamber slide and allowed to adhere overnight. Latex beads-rabbit IgG-FITC complex was added directly to culture medium at a 1:200 dilution and incubated at 37 °C for 2 h. Cells were gently washed with assay buffer twice, followed by counterstaining with 40 μM Hoechst 33342 for 10 min at 37 °C. After two washes, cells were visualized at 20× magnification with a microscope (Olympus IX71). The inhibitor of ULK1(SBI-0206965) and NOX2(GSK2795039) use in this study were purchased from MCEMED EXPRESS.

### Nuclear and cytoplasmic protein extraction

RAW 264.7 cells (1 × 10^7^) were incubated with bacteria for indicated time, remove bacteria and wash by PBS for five times. The cells were harvested and nuclear and cytoplasmic protein was extracted by Nuclear and Cytoplasmic Protein Extraction Kit (Beyotime Biotechnology, China).

### RNA immunoprecipitation

RAW 264.7 cells (1 × 10^7^) were stimulated with bacteria for 6 h and harvested and re-suspended in 1.28 M sucrose, 40 mM Tris–HCl at pH 7.5, 20 mM MgCl2, and 4% Triton X-100 for 20 min on ice. Cells were centrifuged at 2500×*g* for 15 min and the cell pellet was lysed in RIP buffer (150 mM KCl, 25 Mm Tris at pH 7.4, 5 mM EDTA, 0.5 mM DTT, 0.5% NP40) and centrifuged at 13,000 rpm for 10 min. Then the rabbit anti-QKI antibody (Bethyl Laboratories) or rabbit IgG (Bethyl Laboratories) was added to the supernatants and incubated for 2 h at 4 °C. Then protein A/G PLUS-Agarose was added and incubated for 1 h at 4 °C. After washing with RIP buffer three times, the complexes were incubated with 0.5 mg/ml proteinase K at 55 °C for 15 min. Trizol (Life Technologies) was added to extract the RNA. Reverse transcription was carried out and real-time PCR was performed using the following specific primers for PI3K-p110β: 5 ′-GATTATGTTGAACTTATTATTC-3′ (forward) and 5 ′-ATATTATATTTGCCCCACCAAT-3′ (reverse). The level of β-actin mRNA in each immunoprecipitation sample was used to normalize to the RIP results.

### RNA fluorescence in situ hybridization

FAM labelled PI3K-p110β probe (5′-TAGAAGATGAACTGCCCCGC-3′) and negative control (5′-TTTCAGATGTAGGCAAGCC-3′) were purchased from Sangon (Shanghai, China). Firstly, cells were cultured and fixed in 4% paraformaldehyde for 20 min at room temperature, followed with PBS washing three times. Cells were permeabilized with proteinase K (20 μg/ml) for 5 min at 4 °C (Beyotime, Shanghai, China), followed with PBS washing three times. Then cells were incubated with SSC buffer (Solarbio, Beijing, China) for 1 h at 37 °C. Next, FAM labeled *PI3K-p110β* probe mix buffer was added in the dark followed with incubated at 55 °C overnight. Remove the probe mix buffer, cells were washed with 2 × SSC buffer for 10 min, 1 × SSC buffer for 10 min, 0.5 × SSC buffer at 37 °C. Cells were gently washed with assay buffer twice, followed by counterstaining with 40 μM DAPI (Sangon, Shanghai, China) for 10 min at 37 °C. After two washes, cells were visualized at 20× magnification with a microscope (Olympus IX71).

### Indirect immunofluorescence analysis

RAW 264.7 cells were grown on coverslips overnight. MRSA were incubated with FITC-conA (Sangon, Shanghai, China) for 30 min, followed with PBS washing three times. Then cells were incubated with MRSA for 1 h. PBS-washed cells were fixed with 4% formaldehyde for 20 min and then permeabilized with 0.1% Triton X-100 (Sangon, Shanghai, China) for 15 min. After blocking with 5% goat serum for 1 h, cells were incubated with 1:1000 dilution of various antibodies overnight. After washing, cells were incubated with a 1:1000 dilution of Day Light 594 conjugated goat anti-rabbit IgG (TermoScientifc) for 30 min and counterstained with DAPI (Sigma, USA) to stain cell nuclei. Microscopy (Olympus IX71) was used for observing cells and Cell Sens imaging software was used to capture the images.

### Transmission electron microscope

Cultured macrophages were fixed in sodium cacodyl ate-buffered (0.1 mol/L, pH 7.4) 2.5% glutaraldehyde solution for 2 h, then rinsed (3 × 10 min) in sodium cacodylate-buffered (0.1 mol/L pH 7.4) 7.5% saccharose and post-fixed in 1% OsO_4_ solution for 1 h. After dehydration in an ethanol gradient (70% ethanol for 20 min, 96% ethanol for 20 min, 100% ethanol for 2 × 20 min), samples were embedded in Durcupan ACM and then were stained with uranyl acetate and lead citrate. Sections were examined under a FEI TECNAI spirit (USA) microscope at 100 kV.

### Cytokine and ROS detection

The level of TNF-α, IL-1β, IL-10 and IL-6 were measured using ELISA kits (Dakewe, Shenzhen, China) according to the manufacturer’s instructions. The intracellular level of reactive oxygen species was determined using 2′, 7′-dichlorofluorescein diacetates (DCFH-DA, Sigma, USA) according the manufacturer’s instruction. In brief, cells (10^6^ cells/well in 6-well plates) were cultured with *S. aureus* for 1 h and incubated with DCFH-DA at 37 °C for 30 min. The green fluorescence of 2′7 − dichlorofluoresce (DCF) was recorded at 515 nm using a FACS Vantage system (Becton–Dickinson, San Jose, CA, USA), and 10 000 events were counted per sample.

### Nanoparticle for QKI siRNA delivery

QKI siRNA was synthesized by Sangon Biotech Company (Shanghai, China). And specific gene silencing nanoparticles named as siQKI-LCP (lipid-coated particles) was achieved as described previously [39].

### Human PBMC and monocytes isolation and analysis

The samples of healthy controls and Patients with sepsis were collected from Xi Jing hospital of the Fourth Military Medical University (Xi’an, China). Patients with sepsis meeting SIRS criteria (Any 2 of the Following: Heart rate > 90 beats/min, Respiratory rate, beats/min > 20, Temperature > 38 or < 36 °C, White blood cell count > 12,000/mm3 or 10% bandemia).Written informed consent from a next-of-kin was required for enrolment. Retrospective consent was obtained from patients, if possible.

According to the clinical observation and diagnosis, inclusion criteria were proven bacterial infection, together with a systemic inflammatory response (two signs or more among increased heart rate, abnormal body temperature, increased respiratory rate and abnormal white-cell count) and acute organ dysfunction and/or shock.

EDTA-anticoagulated blood samples were collected after primary infection in septic patients. Control samples were collected from matched healthy blood donors (age ± 10 years, sex, race).

Circulating PBMC and monocytes were isolated with Human monocytes separation medium kit (Sangon Biotech, China), and monocytes were harvested by removing non-adhesion cells. After washing with PBS several times, RNA of monocytes and PBMC was extracted, followed with Real-time PCR.

## Supplementary Information

**Additional file 1: Figure S1.** Myeloid QKI deficiency is protective against CLP induced infection (**A**) The survival rate of mice was recorded in cecal ligation and puncture (CLP) induced sepsis model. (**B**) The body temperature of mice within 12 hours was measured by rectal thermometer after CLP induced model carried. (**C-D**) (c) Organs of mice were collected and homogenized after CLP induced model carried. (d) Enumeration of forming bacteria units (c.f.u) from various organs (Blood, kidney, spleen, liver) were analyzed. (**E**) Cytokines (TNF-α, IL-6) level in blood were analyzed by ELISA method after infection in 24hr. (**F**) The peritoneal macrophages were harvested and co-cultured with bacteria at ratio of 1:1, 1:10, 1:100, the control was not infected. After 6 hours, the mRNA expression of TNF-α and PI3K-p110β were analyzed. The cytokines of TNF-α in the supernatant were analyzed by ELISA method. (**G**) C57BL/B6J mice were infected with MRSA, then the percentage of CD45^+^Ly6g^+^CD11b^+^ neutrophils and CD45^+^F4/80^+^CD11b^+^macrophages were analyzed by flow cytometry after 24 hours and 48 hours. All the bars represented the mean of measurements from four independent experiments, and the error bars indicated ± SD. (A-B) are representative of one experiment (n=11 mice/group). A, *p<0.05 Log-rank (Mantel-Cox) test; B, ***p<0.001, (student’s t test). (C-E) are representative of one experiment, (n= 5 mice/group), *p<0.05, *p<0.01, ***p<0.001 (student’s t test). (F) *p<0.05, **p<0.01, ***p<0.001, (student’s t test). (G) are representative of one experiment (n=3 mice/group).

**Additional file 2:** **Figure S2.** QKI deficiency promoted phagocytosis and bactericidal ability of macrophage. (**A**) The number of bacteria units forming after peritoneal cells of QKI^fl/fl^ and LysM^+^QKI^fl/fl^ mice incubated MRSA after 1h and 8h. Representative graph of bacteria forming units were showed. (**B**) The number of bacteria units forming after QKI silenced (shQKI) and scramble (SC) RAW264.7 cells incubated MRSA after 1h and 8h respectively. Representative graph of bacteria forming units were showed. (**C**) Intracellular ROS production was analyzed by flow cytometry analysis after peritoneal cells were incubated with MRSA and dyed with DCFH-DA. The quantification of mean fluorescence intensity (MFI) was shown. (**D**) Expression level of cytokines (IL-6, IL-10, IL-1β and TNF-α) were measured by Enzyme-linked immunosorbent assay (ELISA) in QKI silenced (shQKI) and scramble (SC) RAW264.7 cells incubated with MRSA for 48 hours. (**E**) “Phagocytosis assay” was used to determine the effect of 3-MA to phagocytosis ability of QKI silenced (ShQKI) and scramble (SC) RAW264.7 cells after treated with MRSA and 3-MA simultaneously. The number of bacteria units forming were calculated after 8 hours. Representative graph of bacteria forming units were showed. (**F**) QKI RAW264.7 cells were knockdown of LC3-II and QKI with siRNA respectively, followed with “Phagocytosis assay” to determine the phagocytosis ability of macrophages. (**G and I**) RAW264.7 cells were infected with siRNA for Msl2 and Otulin respectively and NC for control. Cell lysates were subjected to western blot to analyze the protein expression of Msl2, Otulin and QKI, with actin as an internal control. One representative immunoblot is shown (on the left). Graphs (in the middle and right) are representative quantification of the band intensity for immunoblots from three independent experiments. (**H**) RAW264.7 cells were transfected with Flag-tagged QKI vector and the whole-cell lysates were subjected to immunoprecipitation using anti-Flag under denaturing conditions, and immunoblotted with indicated antibodies (EDC3 and DDX6). (**J**) RAW264.7 cells were knockdown of Rnf6 and QKI with siRNA respectively, followed with “Phagocytosis assay” to determine the phagocytosis ability of macrophages. (**K**) Western blot was used to verify the efficiency of siRNA against QKI. Graphs (in the right) are representative quantification of the band intensity for immunoblots from three independent experiments. (**L**) Western blot was used to verify the efficiency of siRNA against LC3-II. Graphs (in the right) are representative quantification of the band intensity for immunoblots from three independent experiments. (**M**) RAW264.7 cells were treated with inhibitor of NOX2(GSK2795039) and ULK-1(SBI-2026965) respectively, followed with “Phagocytosis assay” to determine the phagocytosis ability of macrophages. The number of bacteria units forming were calculated after 8 hours. Enumeration of forming bacteria units (c.f.u) were shown. All the bars represented the mean of measurements from three independent experiments, and the error bars indicated ± SD. (A-C,E-F,J) are representative of one experiment (n= 3 mice/group). ***p<0.001, one-way ANOVA with Tukey’s multiple comparisons test. (D) n= 3 wells/group, *p<0.05, *p<0.01 (student’s t test). (G-I,K,L) are representative of three experiments, ***p<0.001, not significant (ns), one-way ANOVA with Tukey’s multiple comparisons test.

**Additional file 3:** Analysis of clinical and microbiological characteristics of patients with septic shock

**Additional file 4: **Original data sheet

**Additional file 5: **Original data of QKI-interacted proteins analyzed by mass spectrometry

## Data Availability

All data generated or analyzed during this study are included in this published article and its supplementary information files.

## Abbreviations

ATG-5: Autophagy-related gene 5
Becn1: Beclin 1
BMDM: Bone marrow-derived macrophage
CLP: Cecal ligation and puncture
EDC4: Enhancer of mRNA decapping 4
L-10: Interleukin 10
IL-1β: Interleukin 1β
IL-6: Interleukin 6
LC3: Microtubule-associated protein 1 light chain 3
LCP: Lipid coated particles
mTOR: Mechanistic target of rapamycin kinase
PBMC: Peripheral blood mononuclear cell
PCT: Procalcitonin
PI3K: Phosphoinositide 3-kinases
PI3Kp110-α: Phosphoinositide 3-kinase p110-α
PI3Kp110-β: Phosphoinositide 3-kinase p110-β
PI3Kp110-δ: Phosphoinositide 3-kinasep110-δ
QKI: Quaking
QRE: Quaking response element
RNF6: Ring finger protein
RBP: RNA binding protein
TNF-α: Tumor necrosis factor-α
TLR: Toll-like receptor
Ub: Ubiquitin
Vps15: Phosphoinositide-3-kinase regulatory subunit 4
Vps34: Phosphatidylinositol 3-kinase catalytic subunit type 3
