# Supplementary figures and images for: QKI degradation in macrophage by RNF6 protects mice from MRSA infection via enhancing PI3K p110β dependent autophagy
Source: Cell Biosci. 2022 Sep 10;12:154. doi: 10.1186/s13578-022-00865-9 (PMC9464412; doi:10.1186/s13578-022-00865-9)

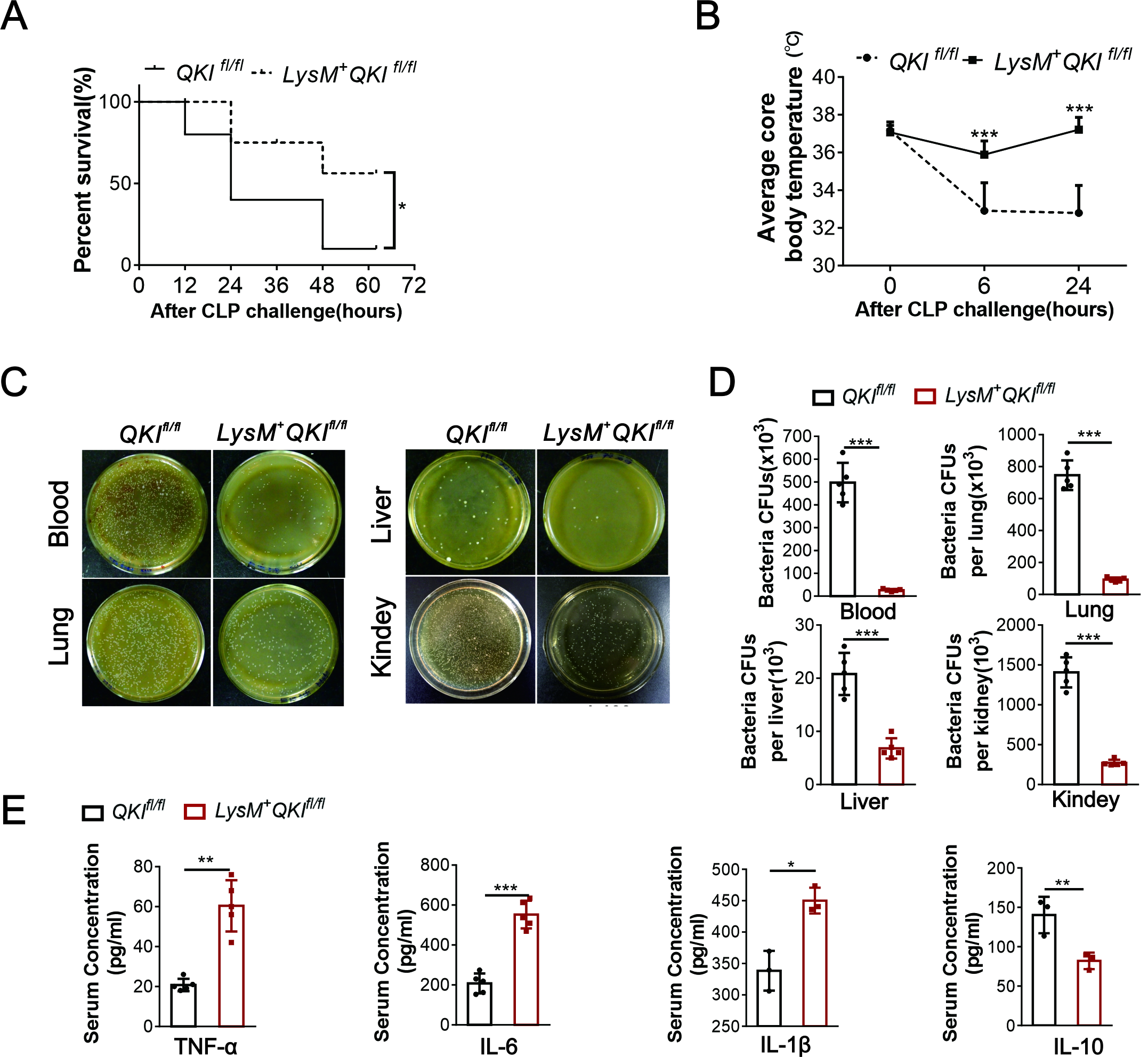

Supplement: Supplementary file 1 — Additional file 1: Figure S1. Myeloid QKI deficiency is protective against CLP induced infection (A) The survival rate of mice was recorded in cecal ligation and puncture (CLP) induced sepsis model. (B) The body temperature of mice within 12 hours was measured by rectal thermometer after CLP induced model carried. (C-D) (c) Organs of mice were collected and homogenized after CLP induced model carried. (d) Enumeration of forming bacteria units (c.f.u) from various organs (Blood, kidney, spleen, liver) were analyzed. (E) Cytokines (TNF-α, IL-6) level in blood were analyzed by ELISA method after infection in 24hr. (F) The peritoneal macrophages were harvested and co-cultured with bacteria at ratio of 1:1, 1:10, 1:100, the control was not infected. After 6 hours, the mRNA expression of TNF-α and PI3K-p110β were analyzed. The cytokines of TNF-α in the supernatant were analyzed by ELISA method. (G) C57BL/B6J mice were infected with MRSA, then the percentage of CD45+Ly6g+CD11b+ neutrophils and CD45+F4/80+CD11b+macrophages were analyzed by flow cytometry after 24 hours and 48 hours. All the bars represented the mean of measurements from four independent experiments, and the error bars indicated ± SD. (A-B) are representative of one experiment (n=11 mice/group). A, *p<0.05 Log-rank (Mantel-Cox) test; B, ***p<0.001, (student’s t test). (C-E) are representative of one experiment, (n= 5 mice/group), *p<0.05, *p<0.01, ***p<0.001 (student’s t test). (F) *p<0.05, **p<0.01, ***p<0.001, (student’s t test). (G) are representative of one experiment (n=3 mice/group). [file 13578_2022_865_MOESM1_ESM.tif]
