# Supplementary material for: QKI degradation in macrophage by RNF6 protects mice from MRSA infection via enhancing PI3K p110β dependent autophagy
Source: Cell Biosci. 2022 Sep 10;12:154. doi: 10.1186/s13578-022-00865-9 (PMC9464412; doi:10.1186/s13578-022-00865-9)
Supplement: Supplementary file 3 — Additional file 3: Analysis of clinical and microbiological characteristics of patients with septic shock [file 13578_2022_865_MOESM3_ESM.pdf]

Table S1 related to Fig.1. Analysis of clinical and microbiological characteristics of patients with septic shock

Pt#	SEX	AGE	SEPSIS SOURCE	Identified Pathogen	PCT
1	F	49	Intra-abdominal	*Staphylococcus aureus*	13.2
2	F	64	Intra-abdominal	*Staphylococcus aureus*	16.3
3	M	36	Intra-abdominal		3.3
4	F	51	Skin and Soft Tissue Infection		2.5
5	F	46	Intra-abdominal		17.0
6	M	66	Urinary tract infection	*Staphylococcus aureus*	6.8
7	M	72	Intra-abdominal		5.0
8	F	44	Intra-abdominal	*Staphylococcus aureus*	19.2
9	F	38	Intra-abdominal	*Escherichia coli*	14.9
10	M	62	Intra-abdominal	*Streptococcus pyogenes*	1.9
11	M	46	Urinary tract infection		3.6
12	M	71	Intra-abdominal		9.3
13	M	84	Pneumonia	*Streptococcus pneumoniae*	11.6
14	F	77	Intra-abdominal		4.2
15	F	52	Intra-abdominal		5.1
16	M	47	Intra-abdominal		6.6
17	M	60	Pneumonia	*Pseudomonas aeruginosa*	4.9
18	M	51	Intra-abdominal		2.6

Pt#	SEX	AGE	SEPSIS SOURCE	Identified Pathogen	PCT
19	F	56	Skin and Soft Tissue Infection	*Escherichia coli*	3.0
20	F	62	Intra-abdominal	*Staphylococcus aureus*	21.3
21	M	27	Intra-abdominal		19.2
22	F	42	Skin and Soft Tissue Infection		5.6
23	F	66	Intra-abdominal		7.3
24	M	66	Intra-abdominal	*Staphylococcus aureus*	6.8
25	M	49	Intra-abdominal		5.0
26	F	64	Intra-abdominal	*Staphylococcus aureus*	19.2
27	F	67	Pneumonia	*Escherichia coli*	14.9
28	M	61	Intra-abdominal		1.9
29	M	51	Intra-abdominal	*Staphylococcus aureus*	6.9
30	M	70	Intra-abdominal	*Streptococcus pyogenes*	11.9
31	M	80	Pneumonia	*Streptococcus pneumoniae*	13.2
34	M	33	Intra-abdominal		8.4
36	M	94	Intra-abdominal		11.2
